# Benzylic C–H Oxidation: Recent Advances and Applications in Heterocyclic Synthesis

**DOI:** 10.3390/molecules29246047

**Published:** 2024-12-22

**Authors:** Nonhlelo Majola, Vineet Jeena

**Affiliations:** School of Chemistry and Physics, University of KwaZulu-Natal, Scottsville, Pietermaritzburg 3209, South Africa; 216047087@stu.ukzn.ac.za

**Keywords:** benzylic C–H oxidation, alkylarenes, ketones, heterocycles, quinoxalines, imidazoles, oxazoles, review

## Abstract

Benzylic C–H oxidation to form carbonyl compounds, such as ketones, is a fundamental transformation in organic synthesis as it allows for the preparation of versatile intermediates. In this review, we highlight the synthesis of aromatic ketones via catalytic, electrochemical, and photochemical oxidation of alkylarenes using different catalysts and oxidants in the past 5 years. Additionally, we also discuss the synthesis of heterocyclic molecules using benzylic C–H oxidation as a key step. These methods can potentially be used in medicinal, synthetic, and inorganic chemistry.

## 1. Introduction

**Figure 1:**
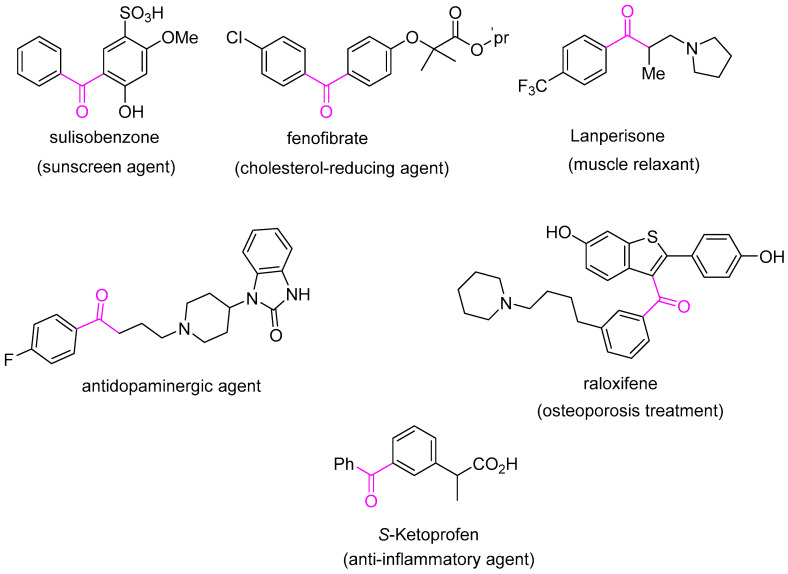
Biologically active compounds comprising aromatic ketone scaffold.

The oxidation of benzylic methylene groups to corresponding carbonyls is one of the fundamental methods for C–H functionalization [1] due to this transformation allowing for the production of high-value products and important intermediates from readily available precursors [2]. The selective oxidation of benzylic compounds into value-added ketones is of considerable importance because they are extensively used as raw materials in the synthesis of many fine chemicals such as fenofibrate, which is used as a cholesterol-reducing agent; lanperisone, which is used as a muscle relaxant; antidopaminergic agents; sulisobenzone (sunscreen agent), which is used as an absorber of UVB and UVA light; raloxifene, which is used in osteoporosis treatment; and *S*-ketoprofen, which is used as an anti-inflammatory agent (Figure 1) [3], to name but a few. Moreover, aromatic ketones are used in the synthesis of perfumes, insecticides, as well as photoinitiators [4,5,6].

The selective oxidation of C–H bonds of alkylarenes to high-value chemicals using environmentally benign oxidants represents a significant achievement in industrial and research fields [7,8,9]. Traditionally, aromatic ketones are synthesized by Friedel–Crafts acylation [10,11] or transition-metal catalyzed methods [12]. Consequently, a plethora of innovative methodologies for benzylic C–H oxidation of alkylarenes to ketones have been documented by different research groups globally. Furthermore, benzylic C–H oxidation has been shown as a key step in the synthesis of various heterocyclic compounds.

**Scheme 1:**
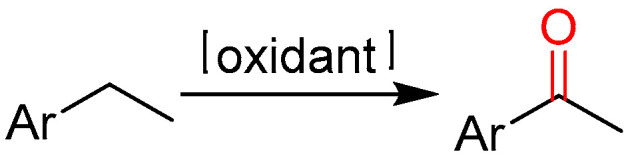
Oxidation of alkylarene [13,14].

Given the importance of this research area, previous reviews reported the findings until 2020 (Scheme 1) [13,14]. Thus, this review will highlight the latest findings of this fast-growing and ever-emerging area from 2020 to 2024. Moreover, this review will highlight the applications of benzylic C–H oxidation in the synthesis of heterocyclic molecules.

## 2. Review on the Latest Benzylic C–H Oxidations

### 2.1. Metal-Catalyzed Oxidation of Benzylic C–H Bonds

**Scheme 2:**
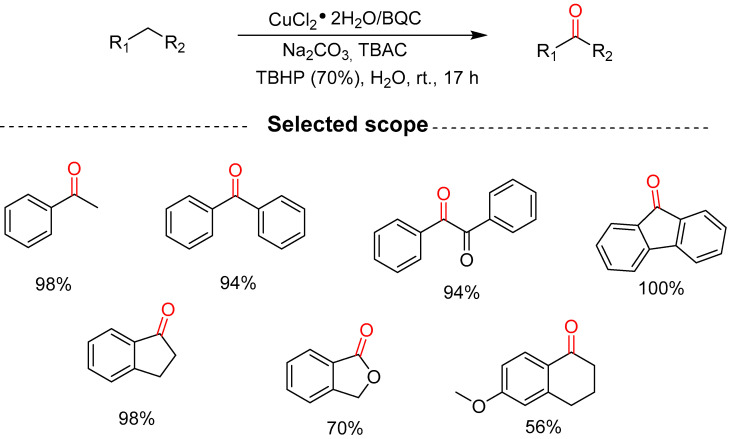
Synthesis of aromatic ketones reported by Ajjou and Rahman.

TBHP in combination with various transition metal catalysts such as Cu, Co, Cr, and Mn has been studied for benzylic oxidation as they are relatively cheap, and their reduced forms can be recycled [15,16,17]. In 2020, Ajjou and Rahman reported the oxidation of alkylarenes with aqueous 70% *tert*-butyl hydroperoxide catalyzed by CuCl_2_·2H_2_O/BQC and the targeted products were obtained in moderate-to-excellent yields of 56–100% (Scheme 2) [18]. This is a highly desirable green system, and the catalyst utilized is water-soluble; however, long reaction times of 17 h are required and the occurrence of side products such as *t*-butylperoxy ethers and benzylic alcohols detracts from this process.

**Scheme 3:**
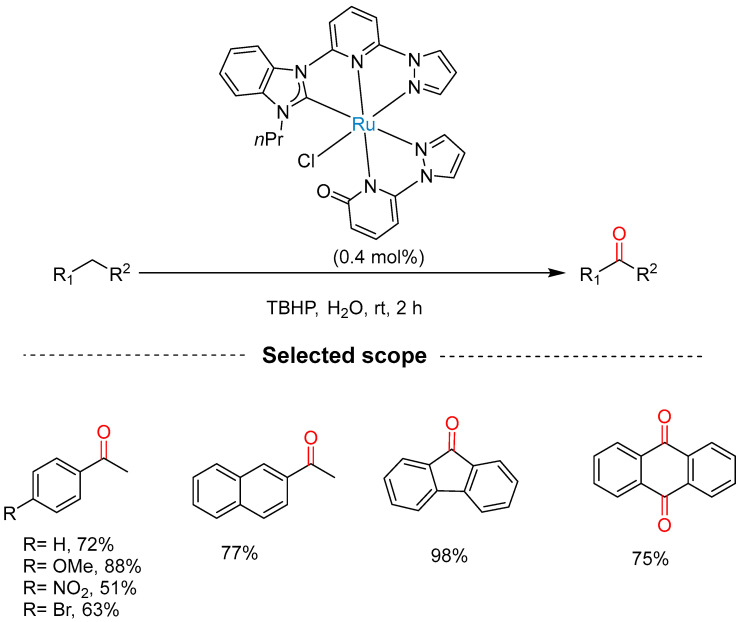
Selected scope for aromatic ketones reported by Liu and co-workers.

Ruthenium-based catalytic systems have emerged as an alternative tool to access ketones via the oxidation of the benzylic C–H bond [19,20]. Coupling ruthenium catalysts with porphyrins-based ligands is well documented in the benzylic C–H oxidation literature [21,22]. Liu and co-workers reported the ruthenium-catalyzed synthesis of aromatic ketones from alkyl arenes and the targeted products were obtained in moderate-to-excellent yields in water at room temperature for 2 h (Scheme 3) [23]. Despite the high cost associated with ruthenium metals, substrates bearing electron-donating groups in *para*-position were obtained in high yields, whereas moderate yields were obtained for substrates bearing electron-withdrawing groups. However, this system uses non-readily available catalysts which must be prepared at high temperatures of 170 °C and long reaction times of 12 h, which detracts from the process.

**Scheme 4:**
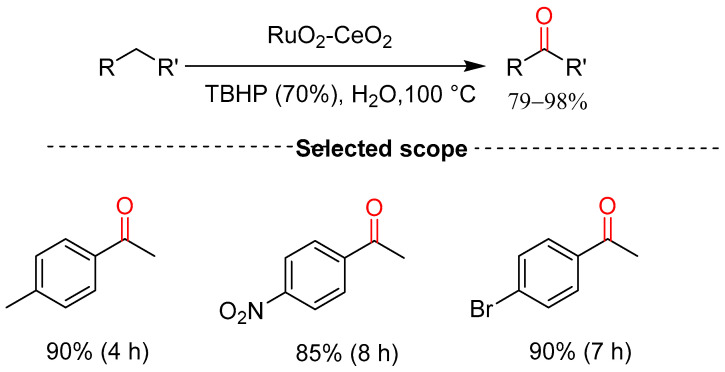
Alkylarene oxidation reported by Zhao and co-workers.

Zhao and co-workers reported the oxidation of alkylarenes to ketones using RuO_2_ supported on MOF-derived CeO_2_ as a catalyst in water in the presence of TBHP at room temperature (Scheme 4) [19]. The authors investigated substrate and scope limitations, and 16 examples of corresponding ketones were obtained in excellent yields of 79–98%. The substrates bearing electron-donating and some electron-withdrawing groups on the benzene ring gave targeted compounds in good-to-excellent yields of 85–90%, whereas those bearing electron-withdrawing nitro- and bromo- on *para*-position required prolonged reaction times for a complete reaction, suggesting that the electronic effect of substituents on the benzene ring affects the rate of oxidation.

**Scheme 5:**
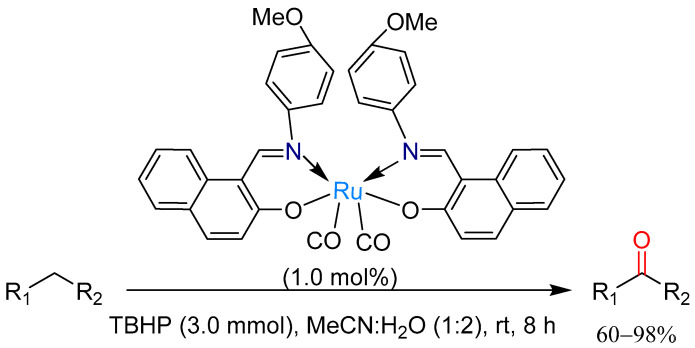
Alkylarene oxidation reported by Hao and co-workers.

Hao and co-workers recently reported the use of ruthenium (II) carbonyl complexes bearing Schiff-base ligands as catalysts in the benzylic C–H oxidation of alkylarenes in the presence of TBHP in an acetonitrile/water solvent system at room temperature for 8 h (Scheme 5) [20]. Up to 20 examples of aromatic ketones were prepared using this easily accessible catalyst which has high catalytic activity, and targeted products were obtained in moderate to excellent (60–98%) isolated yields. This methodology was applicable to a broader substrate scope as even heterocyclic substrates were examined, furnishing the corresponding ketones in good yields (74–83%).

**Scheme 6:**
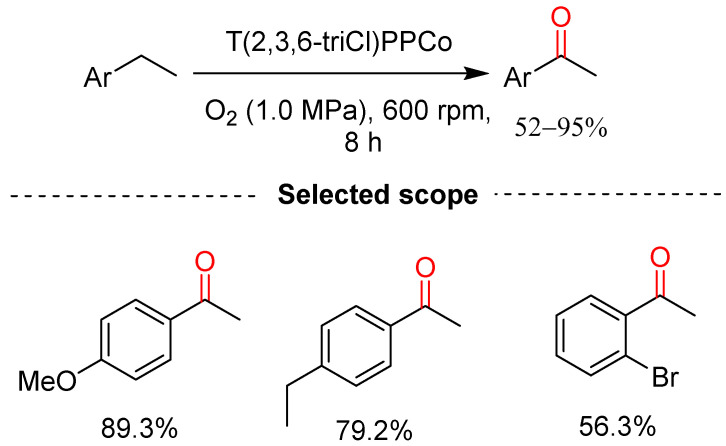
Oxidation of alkylarenes reported by Shen and co-workers.

**Figure 2:**
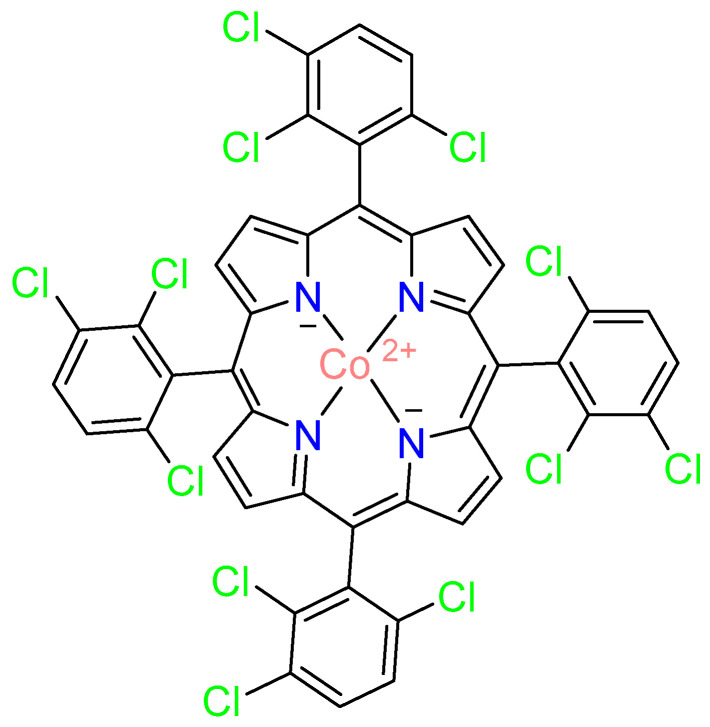
Structure of 5,10,15,20-Tetrakis(2,3,6-trichlorophenyl)porphyrin Cobalt(II).

Most previously documented secondary benzylic C–H oxidations do not conform to green chemistry principles due to heavy solvent corrosiveness and laborious catalyst recovery [21]. To eradicate this, Shen and co-workers reported the solvent-free oxidation of benzylic C–H bonds to aromatic ketones using metalloporphyrins as catalysts and molecular oxygen as an oxidant (Scheme 6) [22], and the selectivity to the corresponding ketones ranged from 52 to 95%. It was observed that bulkier substituents formed plane structures with metalloporphyrin and facilitated the approach of the substrate to the metal center whilst avoiding the attack of active species to metalloporphyrin, thus stabilizing the catalyst (Figure 2). Although this system is solvent- and additive-free and it utilizes simple and easily accessible catalysts, the occurrence of side products such as benzoic acids and benzyl alcohols represents a drawback of this method.

**Scheme 7:**
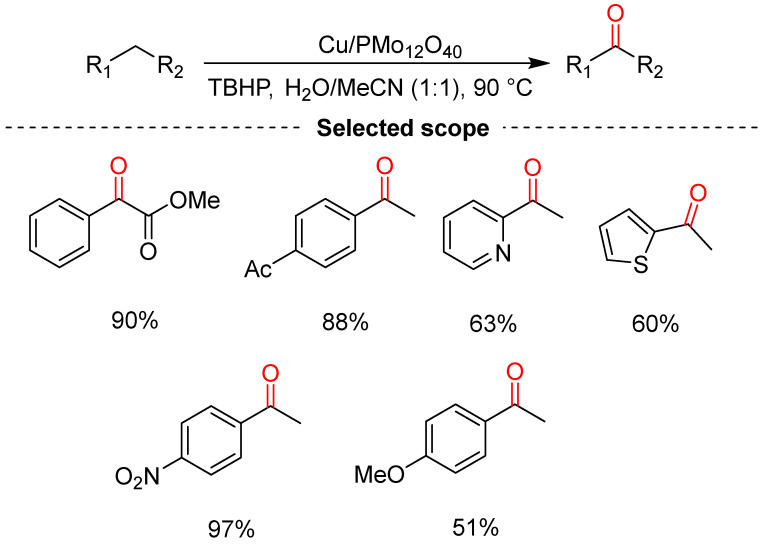
Alkylarene oxidation to aromatic ketones reported by Duan and co-workers.

Duan and co-workers reported the oxidation of alkylarenes to aromatic ketones catalyzed by Cu/PMo_12_O_40_ in the presence of TBHP in water and acetonitrile at 90 °C for 10 h (Scheme 7) [23]. The authors investigated substrate and scope limitations, and the targeted ketones were obtained in moderate-to-good yields (51–97%). The substrates bearing electron-withdrawing groups such as -NO_2_ were more reactive than those bearing electron-donating groups.

Many research groups do not carry out a full substrate scope and limitations study but rather pay attention to the synthesis of a catalyst and its application to a singular example. The catalytic oxidation of ethylbenzene to acetophenone using molecular oxygen or TBHP as an oxidant is well documented in the literature by different research groups investigating the activities of different catalysts (Table 1). Shi and co-workers investigated the activity of 5Co/Ph-HMS catalysts in the oxidation of ethylbenzene at 393 K for 6 h using molecular oxygen as an oxidant, whereby a 55.1% conversion was observed with 86.1% acetophenone selectivity (Table 1, entry 1) [24]. The *N*-hydroxy-1,6-methano[10]-annulene-3,4-dicarboximide/Co(OAC)_2_ catalytic system was then used in the alkylarene oxidation by Zuo and co-workers, whereby 75% conversion of ethylbenzene with 63% acetophenone selectivity was obtained at 6 h (Table 1, entry 2) [25]. Chaudhary and Sharma investigated the activity of the Co-Cu/SAPS-15 catalyst where ethylbenzene was oxidized to acetophenone using TBHP as an oxidant at 100 °C for 6 h and ethylbenzene conversion was obtained to be 96.54% and acetophenone selectivity was 99.77% (Table 1, entry 3) [26]. A palladium-based catalyst, Pd/CeO_2_, was investigated by Kalita and Saikia, whereby a 99.8% conversion was obtained with 79% acetophenone selectivity (Table 1, entry 4) [27]. Liu and co-workers investigated the catalytic activity of Mn-ZSM-5-50 in ethylbenzene oxidation at 80 °C for 6 h, whereby a 43.6% conversion was obtained with 68.8% acetophenone selectivity (Table 1, entry 5) [28]. While these systems have interesting results, it would be useful to conduct a full study and test the catalysts on a variety of substrates to determine the scope and limitations.

**Scheme 8:**
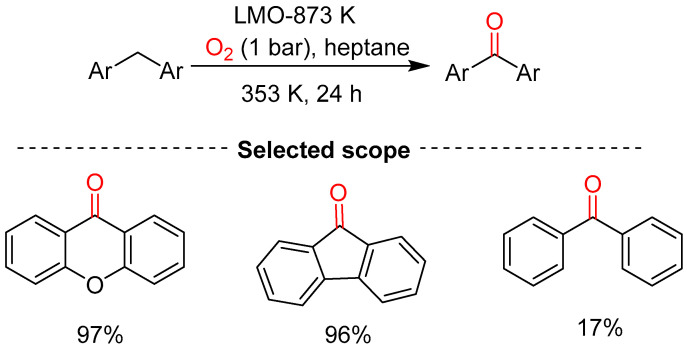
Alkylarene oxidation to aromatic ketones reported by Ozensoy and co-workers.

Among various catalytic processes reported for C–H oxidations, those that utilize nonprecious transition-metal complexes as catalysts and molecular oxygen are attractive for environmental and economic reasons [29,30]. Perovskites have been demonstrated as effective heterogeneous catalysts in various oxidation reactions by different research groups [31,32,33]. In 2021, Ozensoy and co-workers reported the alkylarene oxidation to ketones using the LaMnO_3_ perovskite catalyst (Scheme 8) [34]. The catalytic system was applied to only three substrates, namely xanthene, 9*H*-fluorene, and diphenylmethane, and the corresponding ketones were obtained in yields of 17–99%.

**Scheme 9:**
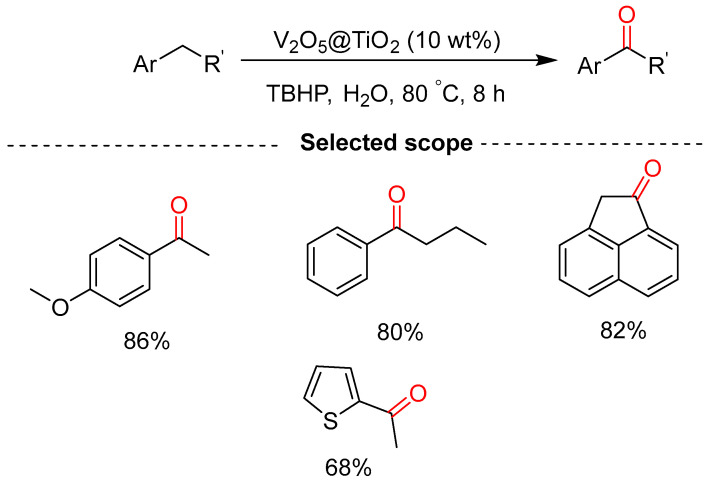
Alkylarene oxidation to aromatic ketones reported by Maurya and co-workers.

Maurya and co-workers reported the green selective oxidation of alkylbenzenes to ketones using a recyclable heterogenous catalyst, V_2_O_5_@TiO_2_ (Scheme 9) [35]. This was applied to a wide range of substrates and provided moderate-to-good yields of 68–94%. However, the use of stochiometric amounts of TBHP and non-commercially available catalysts which must be prepared at high temperatures for longer reaction times detracts from this method.

**Scheme 10:**
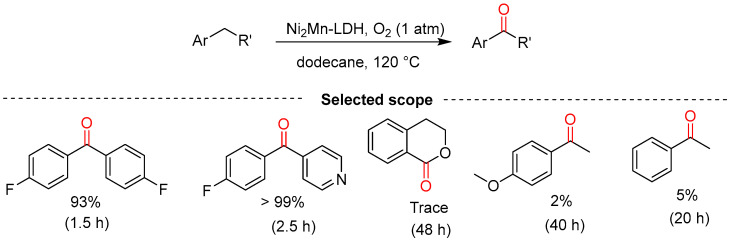
Alkylarene oxidation to aromatic ketones reported by Zhou and co-workers.

Zhou and co-workers reported the preparation of aromatic ketones from alkylarenes using the Ni_2_Mn-LDH as a catalyst and molecular oxygen as an oxidant in dodecane at 120 °C (Scheme 10) [36]. This system was highly substrate-specific as disubstituted substrates produced high yields in short reaction times, whereas monosubstituted substrates produced diminished yields even after prolonged reaction times at higher temperatures.

**Scheme 11:**
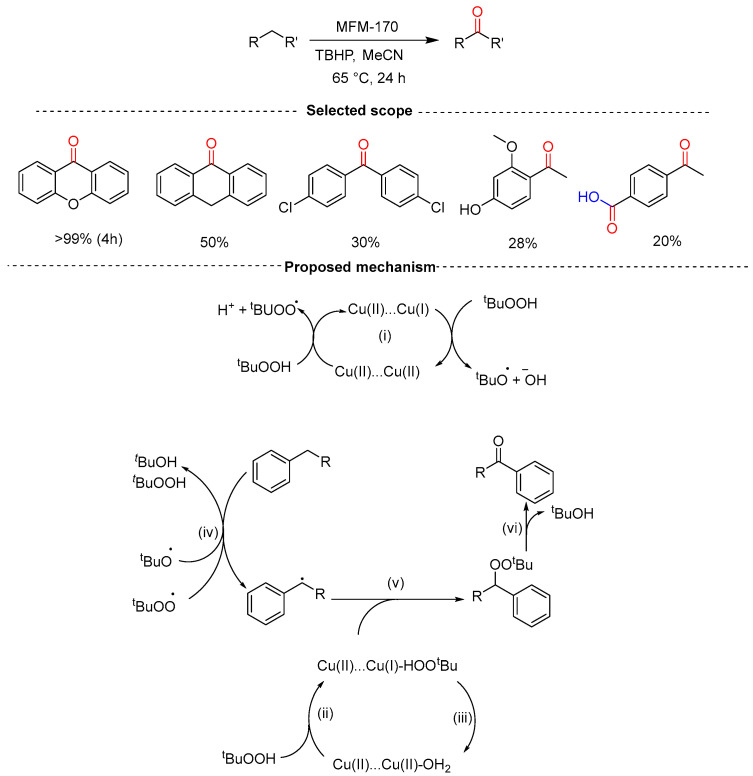
Alkylarene oxidation to aromatic ketones reported by Tuna and co-workers.

Tuna and co-workers reported the benzylic C–H oxidation of alkylarenes to corresponding aromatic ketones using MFM-170 in the presence of TBHP in acetonitrile at 65 °C for 24 h (Scheme 11) [37]. The authors tested their catalytic system on a broad range of substrates to determine its scope and limitations where bulkier substrates displayed high yields were compared to less bulkier substrates. Substrates with oxidatively sensitive groups such as -OH and -CHO were also tested, whereby the hydroxy group was retained, and the aldehyde group was oxidized to a carboxylic acid. The authors then proposed a viable mechanism after an in-depth EPR spectroscopic study and SPXRD was conducted and provided evidence for the important redox behavior of [Cu_2_(O_2_CR)_4_] which drives the reaction.

**Scheme 12:**
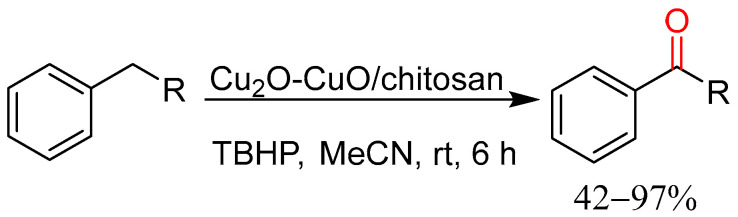
Alkylarene oxidation to aromatic ketones reported by Tantirungrotechai and co-workers.

Tantirungrotechai and co-workers built upon that study and used a copper–oxide chitosan composite as a heterogeneous catalyst for the benzylic C–H oxidation of alkylarenes to ketones at room temperature for 6 h, and the corresponding ketones were obtained in moderate-to-good yields of 42–97% (Scheme 12) [38]. The gram-scale reaction of fluorene afforded 88% yield of fluorenone, similar to that of the small-scale reaction, suggesting that this is an effective method for scale-up applications.

**Scheme 13:**
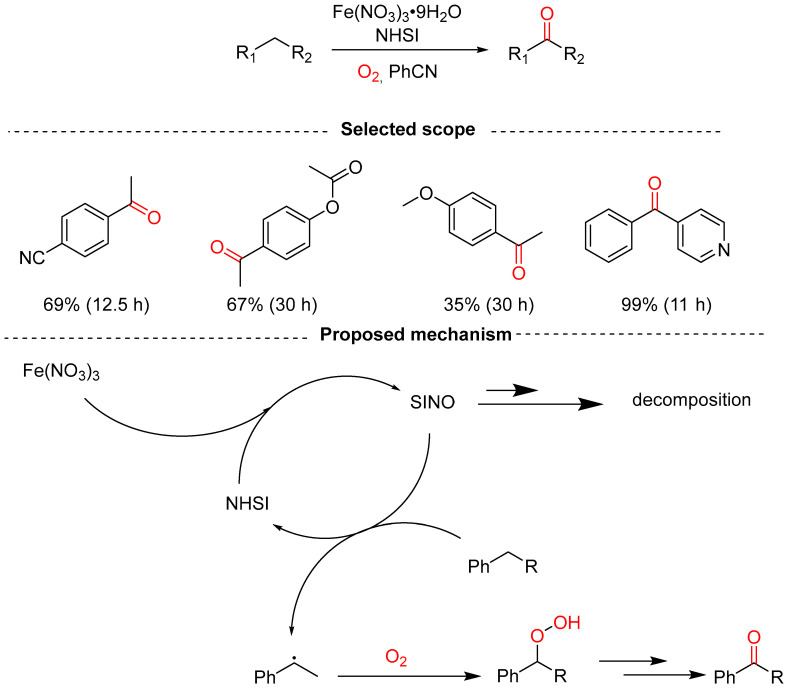
Alkylarene oxidation reported by Yin and co-workers.

N-Hydroxyimides (NHIs) are one of the most promising organocatalysts for benzylic aerobic oxidation as it has many merits i.e., easy preparation and good catalytic performance. Yin and co-workers documented the aerobic oxidation of alkylarenes to aromatic ketones using N-hydroximide organocatalyst, Fe(NO_3_)_3_·9H_2_O/NHSI in benzonitrile at 90 °C and the desired products were obtained in moderate to good yields of 33–99% (Scheme 13) [39]. The authors conducted various control experiments to gain insight into the mechanism that is initiated by Fe(NO_3_)_3_ with NHSI. SINO then abstracts a hydrogen atom from the substrate to give a benzyl radical, which is then oxidized to a ketone *via* a peroxy intermediate.

**Scheme 14:**
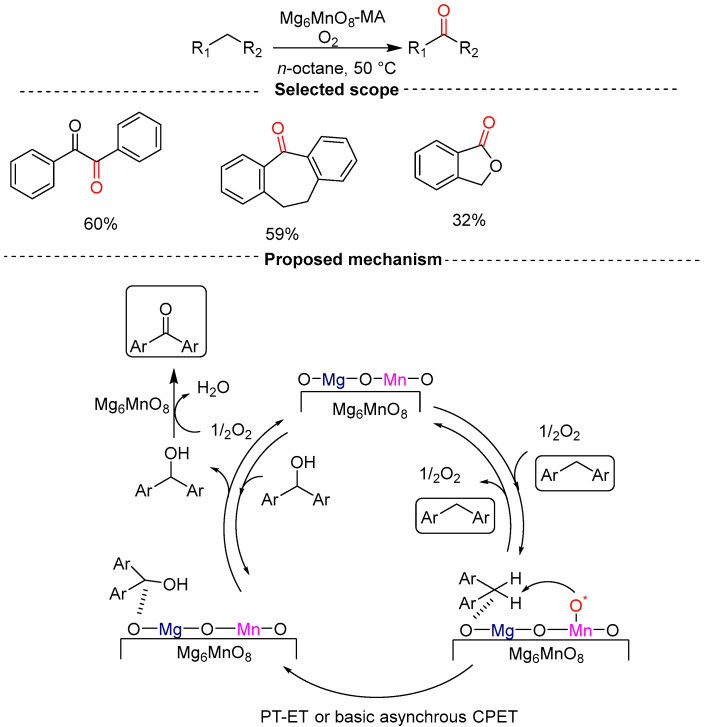
Alkylarene oxidation reported by Hara and co-workers.

Hara and co-workers documented the base-assisted aerobic C–H oxidation of alkylarenes using the Mg_6_MnO_8_ nanoparticle catalyst in octane at 50 °C and the corresponding ketones were obtained in moderate-to-excellent yields of 32–97% (Scheme 14) [40]. The authors proposed a reaction mechanism that commences by the reversible adsorption of a substrate and oxygen at the Mg_6_MnO_8_ surface to form the adsorbed substrate to generate the corresponding adsorbed alcohol followed by reversible desorption of adsorbed oxygenated species. The alcohol undergoes rapid oxidation to furnish the corresponding ketone.

**Scheme 15:**
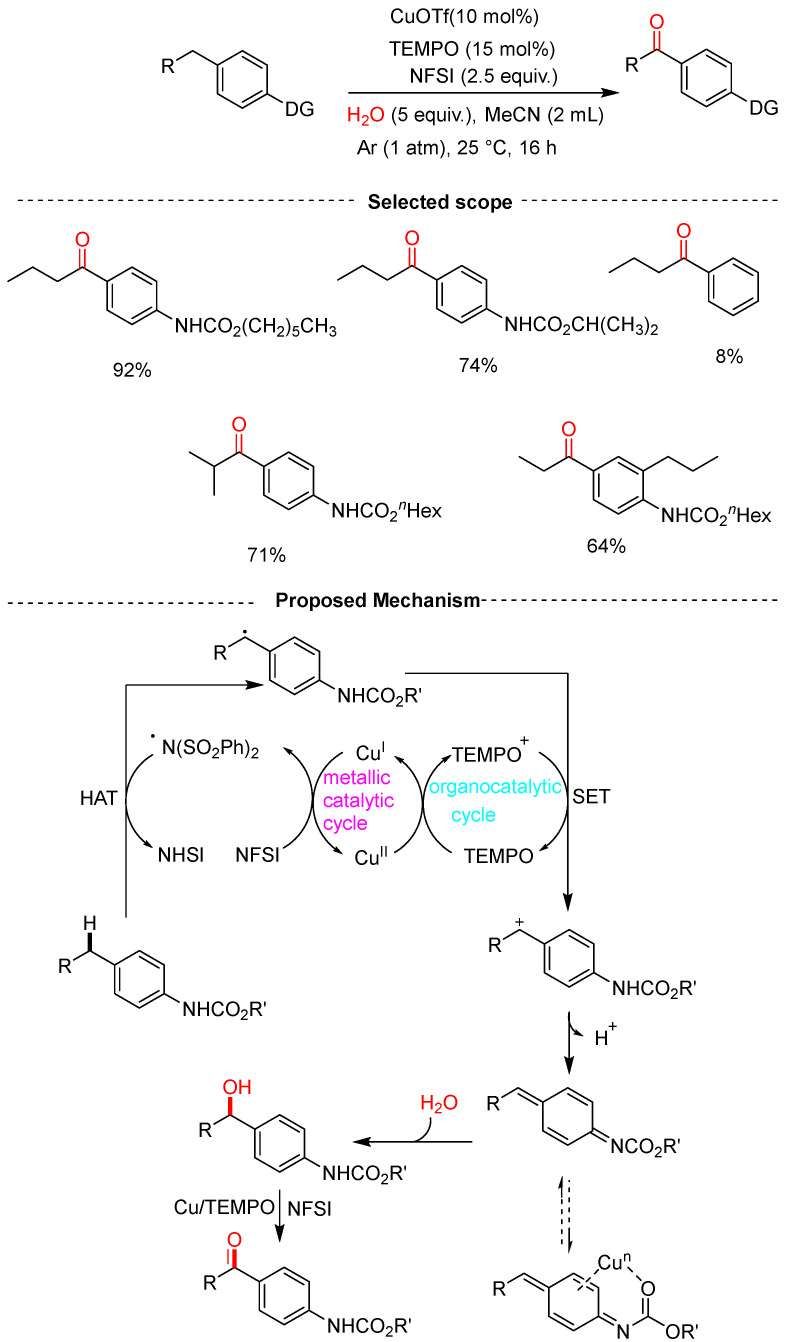
Alkylarene oxidation reported by Chen, Li and co-workers.

Chen, Li and co-workers reported the remote carbamate-directed site-selective benzylic C–H oxygenation of alkylarenes via synergistic copper/TEMPO catalysis at room temperature in acetonitrile using water as an oxygen source at 25 °C for 16 h (Scheme 15) [41]. In the presence of a directing group in the *para*-position of the alkylarene, the corresponding aromatic ketones were obtained in moderate-to-high yields of 42–92%. However, in the absence of a directing group, a diminished yield of 8% was observed, thus suggesting that this system works better for alkylarenes with directing groups. The authors conducted a series of control experiments and kinetic studies to better understand the mechanism which revealed that it commences by the oxidation of the Cu(I) catalyst by NFSI which gives the imidyl NCR A along with Cu(II) species. Substrate and NCR A undergoes an HAT process to give benzyl radical, which is then oxidized by oxonium TEMPO to give benzyl cation that is stabilized by the remote carbamate directing group after tautomerization and dehydrogenation, thus generating methine cyclohexadienyl imide which consequently is trapped by water, providing benzyl alcohol. The targeted product is then furnished by Cu/TEMPO-catalyzed oxidation of benzyl alcohol.

**Scheme 16:**
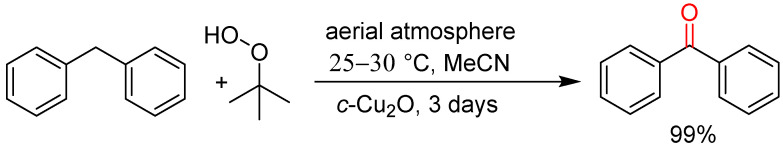
Diphenylmethane oxidation to benzophenone reported by Soni and co-workers.

Soni and co-workers reported the oxidation of diphenylmethane using TBHP as an oxidant in the presence of the *c*-Cu_2_O nanocatalyst in acetonitrile at room temperature for three days and benzophenone was obtained in excellent ~99% yield (Scheme 16) [42].

**Scheme 17:**
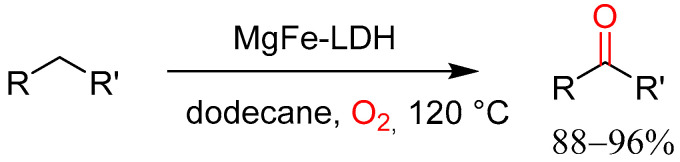
Alkylarene oxidation reported by Peng and co-workers.

Peng and co-workers documented the oxidation of alkylarenes to aromatic ketones using MgFe-LDH as a catalyst in the presence of molecular oxygen in dodecane at 120 °C (Scheme 17) [43]. This methodology was applicable to diaryl-substituted compounds as the corresponding ketones were obtained in excellent yields of 88–96%. However, it would be useful to apply this method to monoaryl-substituted and heterocyclic substrates to determine the scope and its limitations.

**Scheme 18:**
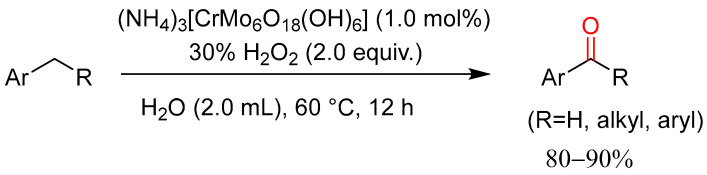
Alkylarene oxidation reported by Yu and co-workers.

Yu and co-workers reported the selective oxidation of alkylarenes in water catalyzed by the polyoxometalate-supported chromium catalyst in the presence of hydrogen peroxide (Scheme 18) [44]. Nineteen examples of aromatic ketones were synthesized in high-to-excellent yields of 80–90% under a nitrogen-rich environment. This catalyst demonstrated high stability as it can be recycled up to six times whilst showing good activity as it can oxidize alkylarenes to various carbonyl compounds including aldehydes, carboxylic acids, etc. However, the use of a non-commercially available catalyst that has to be prepared detracts from this method.

**Scheme 19:**
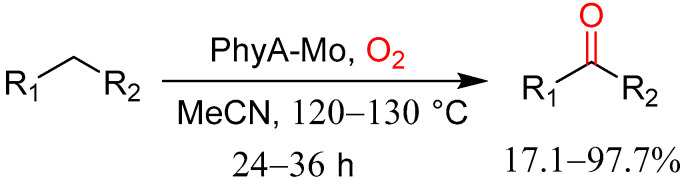
Alkylarene oxidation reported by Song and co-workers.

Song and co-workers reported a bio-derived polyoxometalate hybrid catalyst for the selective oxidation of benzylic C–H bonds which was applicable to a broad substrate scope and was used in the absence of the initiator, and up to ~98% yield of the desired ketones was obtained [45] (Scheme 19). Despite the high yields of ketone, this system utilizes a non-commercially available catalyst and has to be prepared in a tedious three-step process at high temperatures in long reaction times.

**Scheme 20:**
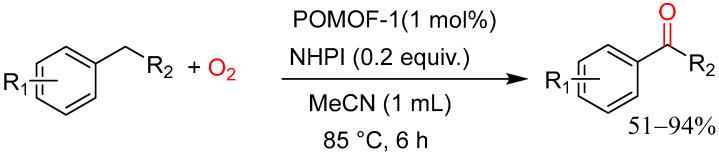
Alkylarene oxidation reported by Zhao, Xuan and co-workers.

Zhao, Xuan and co-workers reported the selective oxidation of benzylic C–H bonds using molecular oxygen in the presence of a polyoxometalate-based metal–organic framework heterogeneous catalyst, POMOF-1, and NHPI in acetonitrile at 85 °C for 6 h (Scheme 20) [46]. The catalyst was synthesized via the Anderson-type reaction and the corresponding ketones were obtained in moderate to high isolated yields of 51–88%.

**Scheme 21:**
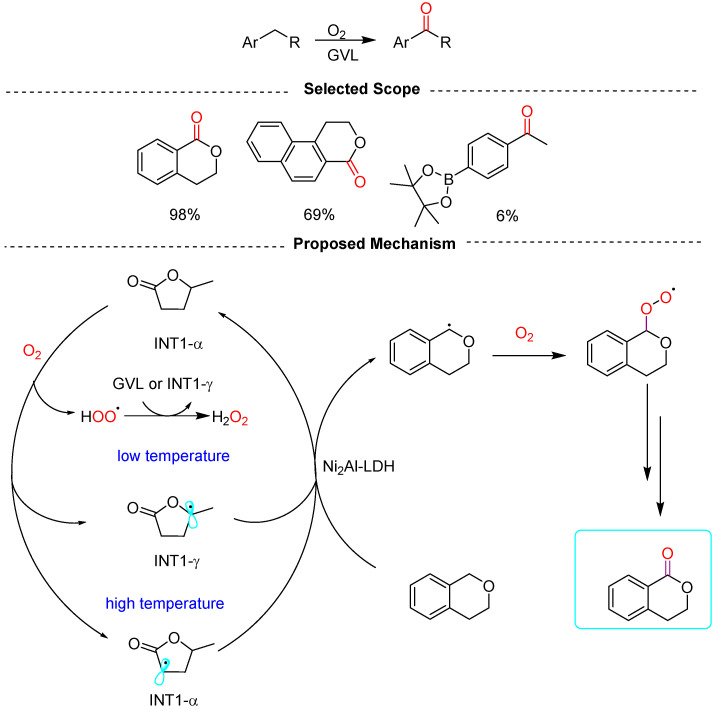
Alkylarene oxidation reported by He and co-workers.

He and co-workers reported the synthesis of ketones from alkylarenes using 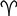-valerolactone (GVL) as a catalyst in the presence of molecular oxygen and the corresponding ketones were obtained in moderate-to-excellent yields of 31–98% (Scheme 21) [47]. However, this system was inapplicable to the boric acid ester-substituted substrate as only 6% of the desired product was obtained. The authors then investigated the use of this method in a gram-scale synthesis in which the corresponding ketone was obtained in 80% yield. The authors then applied this system in the preparation of 7*H*-Dibenzo[*c*,*e*]oxepin-5-one, menthone, and –(-)-Menthyl benzoate. The authors then proposed a reaction mechanism whereby molecular oxygen abstracts the α-proton from GVL under high temperatures and 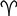-proton under low temperatures to give the corresponding GVL radical and hydroperoxyl free radical. The two radicals from GVL activate the isochroman molecule via HAT selectively with the assistance of Ni_2_Al-LDH. The hydroperoxyl free radical then further reacts with GVL to generate intermediates and hydrogen peroxide. The formed isochroman radicals are rapidly trapped by oxygen to give peroxyl radicals which evolves into the oxidation process [48].

Okuda and co-workers documented the oxidation of alkylbenzenes to ketones and alcohols, using the NB4_Co−Br^+^Br^−^ as the catalyst and oxone and phosphate buffer in acetonitrile at 25 °C for 2 h, and the ketone selectivity ranged from 76 to 96% [49]. Up to eight examples of ketones were synthesized; however, alcohol was obtained as a side product.

### 2.2. Non-Metal Catalyzed Oxidation of Benzylic C–H Bonds

**Scheme 22:**
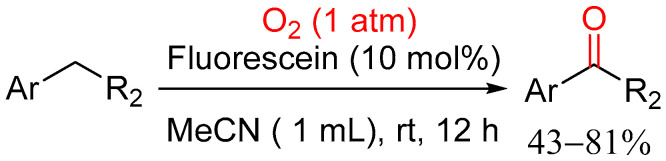
Alkylarene oxidation to aromatic ketones reported by Zhu and co-workers.

Fluorescein has attracted immense attention as an alternative to transition-metal complexes due to it having a similar ground-state excited redox potential to its transition metal counterparts [50,51]. Owing to this, Zhu and co-workers reported the photocatalyzed oxidation of benzylic C–H bonds using fluorescein as a photocatalyst in acetonitrile at room temperature for 12 h (Scheme 22) [52]. Their system was applicable to a broad substrate scope and the corresponding aromatic ketones were obtained in moderate-to-high yields of 43–81%. A gram-scale experiment was performed, and the desired product was obtained at 78% isolated yield, indicating that this is a feasible method for the industrial mass production of aromatic ketones.

**Scheme 23:**
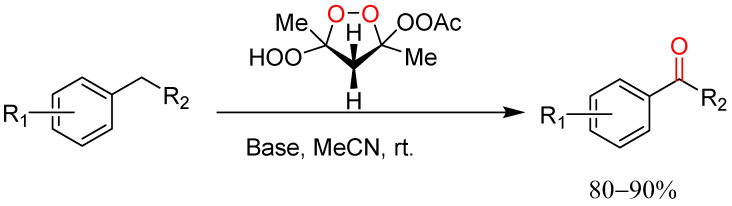
Alkylarene oxidation to aromatic ketones reported by Najminejad.

Najminejad documented the oxidation of alkyl-substituted aromatics with singlet molecular oxygen generated from *trans*-3,5-hydroperoxy-3,5-dimethyl-1,2-dioxolan-3-yl-ethaneperoxate in MeCN at room temperature. The corresponding ketones were obtained in high yields of 80–90% for all substrates including those bearing electron-donating and electron-withdrawing groups (Scheme 23) [53].

**Scheme 24:**
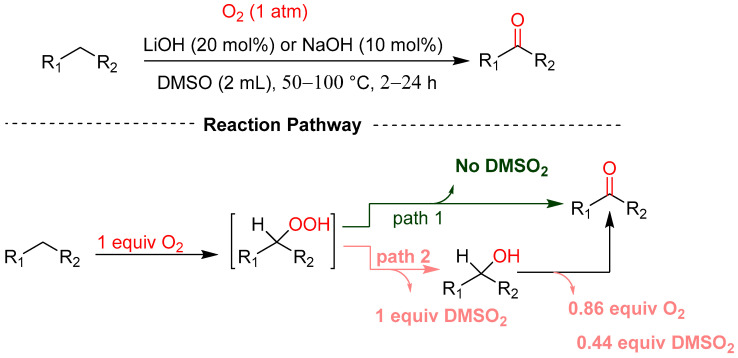
(Hetero)benzylic C–H oxidation reported by Li and co-workers.

Li and co-workers reported the aerobic oxidation of (hetero) benzylic C–H bonds to aromatic ketones using molecular oxygen as an oxidant in the presence of a base in DMSO (Scheme 24) [54]. The ketones were obtained in high yields by choosing an alkalinity-matched inorganic base with the acidity of (hetero)benzylic C–H bonds. The authors proposed a reaction pathway after a series of control experiments were conducted, which revealed that the substrate reacts with a base and molecular oxygen to form a peroxide intermediate which can undergo either dehydration or reduction, and eventually furnish desired ketones in one of two ways.

**Scheme 25:**
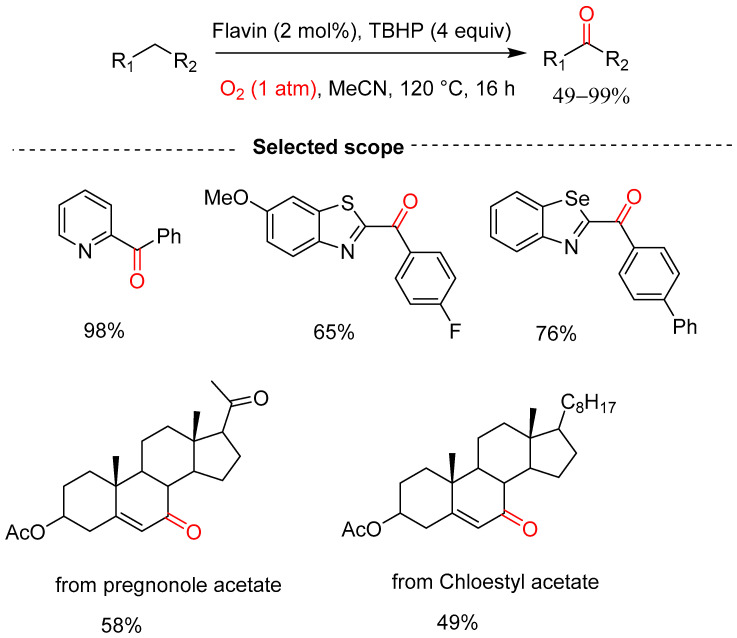
Het(aryl) oxidation reported by Gan and co-workers.

Gan and co-workers reported the het(aryl) alkane oxidation to ketones using flavin in the presence of TBHP under oxygen-rich environment in acetonitrile at 120 °C for 16 h (Scheme 25) [55]. The authors examined the scope and its limitations, which revealed that this system was applicable to a wide range of substrates and the ketones were obtained in moderate-to-excellent yields (49–99%). Various intermediates that are utilized in the preparation of biologically important compounds were synthesized, such as the intermediate from pregnenolone acetate which was furnished with a 58% isolated yield. However, the use of higher temperatures and longer reaction times detracts from this process.

**Scheme 26:**
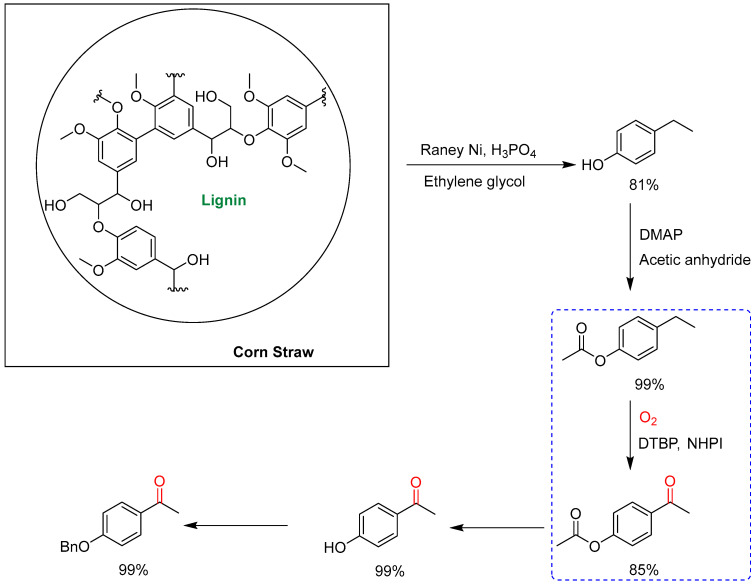
Benzylic C–H oxidation as key intermediate in pharmaceutical compound synthesis from lignin.

Liu and co-workers developed a catalytic strategy for the conversion of lignin to several pharmaceutical intermediates based on selective depolymerization to generate 4-ethylphenol followed by benzylic C–H oxidation with molecular oxygen to furnish 4-acetoxyacetophenone as a key intermediate for the synthesis of various pharmaceutical intermediates (Scheme 26) [56]. Raney nickel and a strong acid was used as a catalyst for the depolymerization and a green benzylic C–H oxidation using environmentally benign O_2_ as the oxidant afforded the key intermediate in 85% yield.

### 2.3. Photocatalytic Oxidation of Benzylic C–H Bonds

**Scheme 27:**
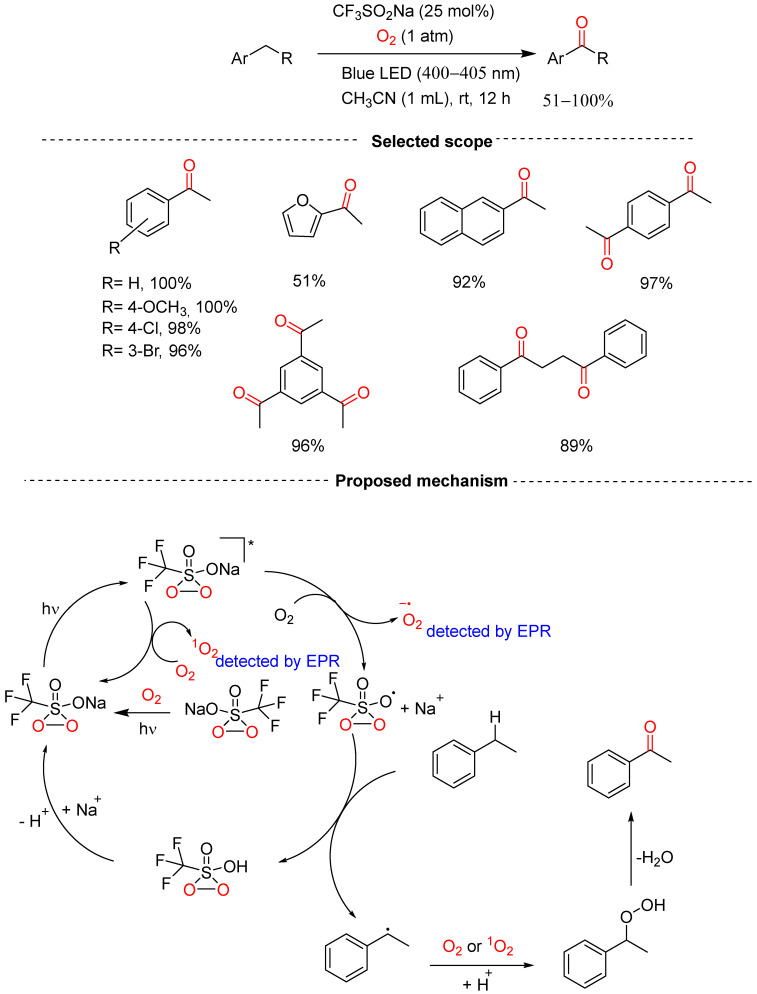
Trifluoromethanesulfinate-mediated synthesis of aryl ketones from alkylarenes by Fu and co-workers. * indicates light activation.

Visible light photocatalysis has received enormous attention in organic synthesis due to its simplicity, economic, and environmental friendliness [57,58]. On that note, Fu and co-workers reported light and oxygen-enabled sodium trifluoromethanesulfinate-mediated selective oxidation of C–H bonds allowing for the preparation of aromatic ketones from readily available alkyl arenes (Scheme 27) [59]. Their methodology was applicable to a wide range of substrates such as neutral, electron-rich, and electron-donating with excellent yields of 95–100% at room temperature for 12 h. This system was also applied to a heterocyclic substrate and produced the corresponding ketone in a moderate 51% yield. Alkyl-containing arenes were also investigated and it was found that the length of the alkyl chain and substituent variation on alkyl did not affect the reactivity of the substrates. The authors proposed a radical mechanistic pathway which was confirmed by electron paramagnetic resonance. The initial light irradiation of the catalyst produces an excited state and an SET of the excited state to oxygen generates two radicals. The radical obtained from the catalyst would then react with the substrate to create a benzyl radical which, upon reaction with oxygen, would give a peroxide which undergoes dehydration to furnish the desired ketones.

Huang and co-workers documented the photocatalysis of benzylic C–H bonds catalyzed by MIL-125(Ti) under visible light to afford the aromatic ketones in selectivities of 95 − >99% in the presence of NHPI [60]. Despite the high selectivity to the ketones, only seven examples were synthesized. Thus, it would be beneficial to apply this system to other substrates such as heterocyclic and bulkier substrates to determine the scope and its limitations.

**Scheme 28:**
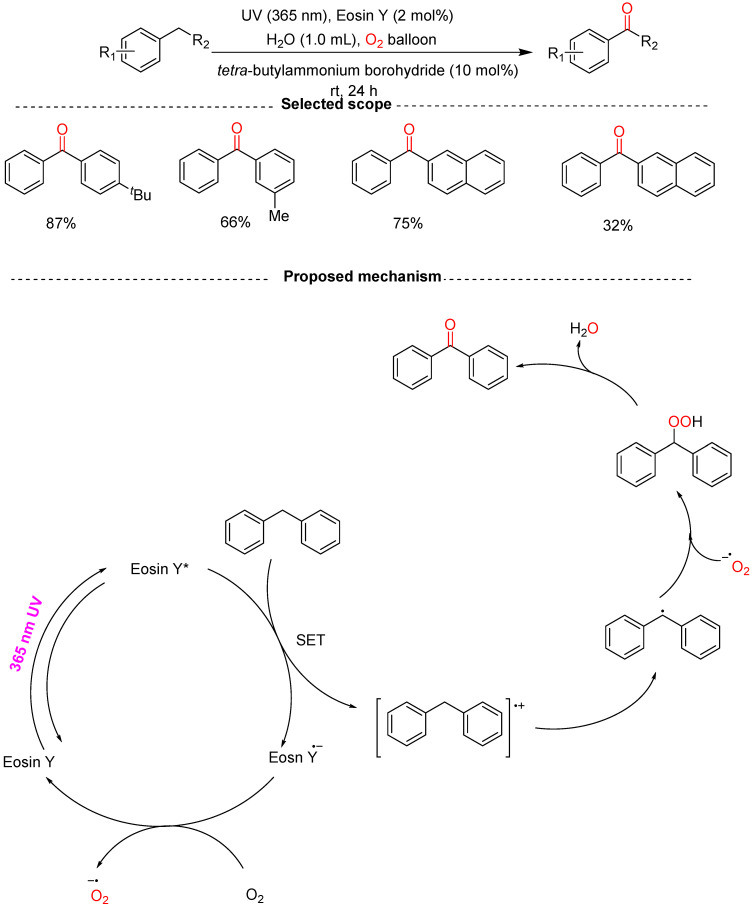
Alkylarene oxidation reported by Duan and co-workers. * indicates activation by light.

Dai and co-workers reported the photocatalytic benzylic oxidation promoted by eosin Y in water to prepare aromatic ketones from alkylarenes in moderate-to-good yields (32–89%) (Scheme 28) in 24 [61]. This was applicable to a broad substrate scope including those bearing electron-donating, electron-withdrawing, and bulkier substrates. This is a highly desirable system as water is utilized as a solvent at room temperature; however, long reaction times are required. After a series of control experiments which revealed that the reaction proceeded via a single electron-transfer pathway, the authors proposed a plausible mechanism. It commences from the photocatalyst Eosin Y which forms the excited species Eosin Y* upon UV irradiation, which then reacts with the substrate via SET to produce free radical cation and Eosin Y^·−^ radical anion followed by dissociation of a hydrogen atom from a free radical cation to produce benzylic radical. The Eosin Y^·−^ can be oxidized by molecular oxygen to form Eosin Y, to form a photocatalysis cycle. Meanwhile, the molecular oxygen is reduced to generate superoxide radical ions which react with the benzylic radical to form a hydrogen peroxide intermediate which then undergoes dehydration to furnish the desired ketone.

Terent’ev and co-workers investigated the catalytic activity of heterogenous photocatalyst TiO_2_/NHPI by performing the benzylic C–H oxidation of ethylbenzene to acetophenone under visible light and molecular oxygen as an oxidant [62]. Despite the high selectivity of the ketone and high catalytic activity shown by the catalyst, this system had side-product occurrences such as the formation of an acid. Additionally, only one example was investigated; therefore, it would be beneficial to test this catalytic system on a wide range of substrates to determine its scope and limitations.

**Scheme 29:**
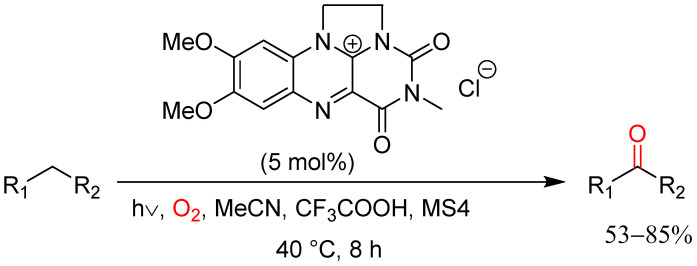
Alkylarene oxidation reported by Sirkosi and co-workers.

Sirkosi and co-workers reported the aerobic oxidation of unactivated benzylic substrates to aromatic ketones under visible light using ethylene-bridged flavinium salts as photocatalysts (Scheme 29) [63]. The substrates were varied to determine the scope and limitations of this methodology and it was applicable to a range of substrates as the corresponding ketones were obtained in 24–85% yields after prolonged reaction times.

**Scheme 30:**
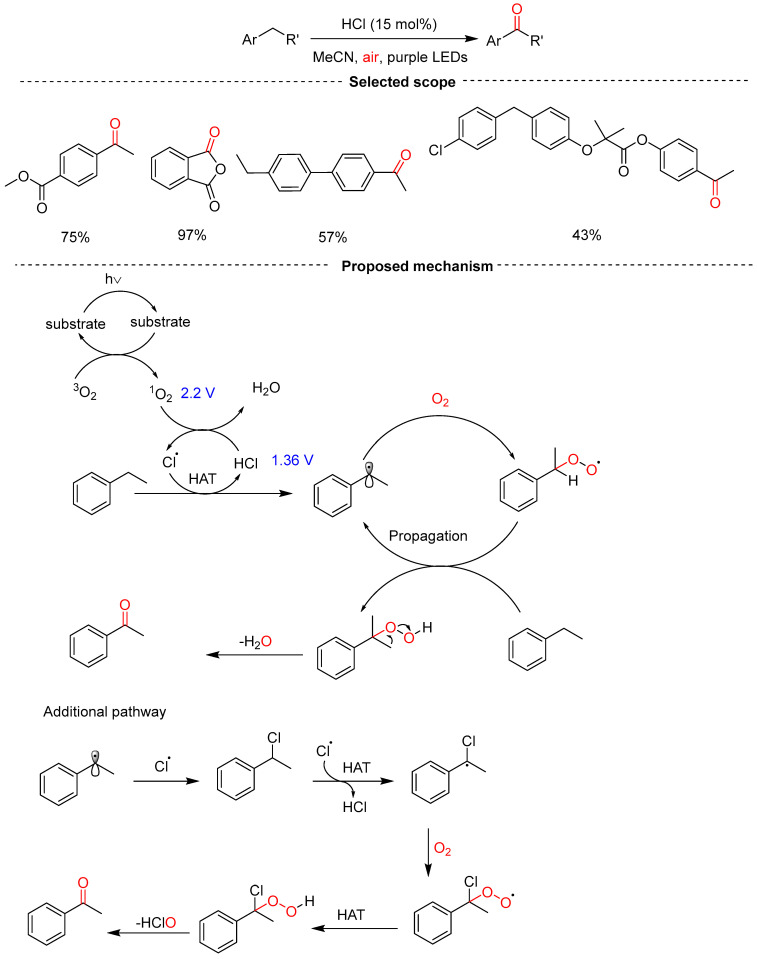
Alkylarene oxidation to aromatic ketones reported by Niu, Wang and co-workers.

Niu, Wang and co-workers reported the HCl-catalyzed aerobic oxidation of alkyl arenes to aromatic ketones in acetonitrile under purple LEDs (Scheme 30) [64]. The corresponding ketones were obtained in moderate yields to excellent yields of 31–99%. This system was also applied to the anti-inflammatory and analgesic drug ibuprofen to give the corresponding ketone in moderate yields of 43%. The authors then investigated the reaction mechanism by conducting a series of studies, whereby TEMPO was used as a radical scavenger and was able to confirm the presence of benzyl radicals. The irradiation of the substrate with visible light produces the excited species substrate* which then transfers energy to O_2_ to generate ^1^O_2_ that reacts with Cl^−^ to give a chlorine radical and water. The chlorine radical undergoes HAT with alkylbenzene to give a benzyl radical which is subsequently trapped by O_2_ to give benzylic peroxyl radical that reacts rapidly with the second alkylbenzene molecule and initiates the radical chain to give benzylic hydroperoxide which undergoes heterolytic fragmentation to furnish the aromatic ketone.

**Scheme 31:**
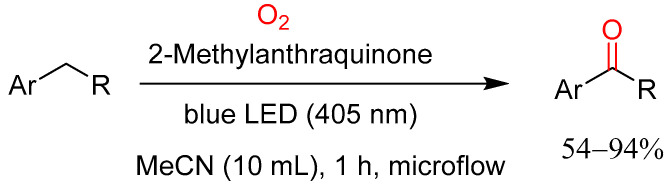
Alkylarene oxidation reported by Chen, Li, Xu and co-workers.

Anthraquinones (AQs) are considered the best photocatalysts because of their high redox potential and high absorption in the UV-visible region [65]. Chen, Li, Xu and co-workers reported the photocatalytic oxidation of C–H bonds based on a microchannel reactor using 2-methylanthraquinone as a photocatalyst in acetonitrile for 1 h and the corresponding ketones were obtained in moderate-to-excellent yields of 54–94% (Scheme 31) [66]. This system works in shorter reaction times in a microflow reactor and uses molecular oxygen as an oxidant, and it is thus economical.

**Scheme 32:**
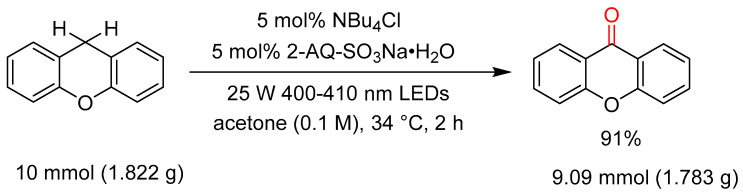
Scale-up synthesis of xanthone reported by Nguyen and co-workers.

Nguyen and co-workers documented the preparation of benzylic ketones from alkylarenes under a 25 W 400–410 nm LED lamp, in the presence of sodium anthraquinone-2-sulfonate monohydrate (2-AQ-SO_3_Na·H_2_O) in acetone in ambient air and the corresponding ketones were obtained in moderate-to-high yields of 49–89%. The authors then investigated whether this system could be scaled up by using xanthene as a model substrate which was converted to xanthone on a 10 mmol scale with excellent yield of 91% (Scheme 32) [67].

**Scheme 33:**
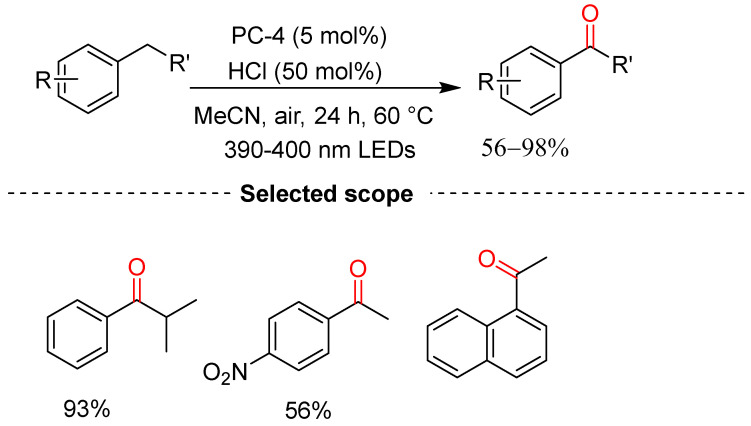
Alkylarene oxidation reported by Xu, Li and co-workers.

Xu, Li and co-workers documented the photocatalytic oxidation of alkylarenes to ketones in the presence of PC-4 and HCl as catalysts using air under 390–400 nm in acetonitrile for 24 h (Scheme 33) [68]. The substrate and scope limitations were investigated whereby neutral substrates gave the targeted ketones in good-to-high yields of 82–93%, substrates bearing electron-rich substituents furnished the desired ketones in 78–91% isolated yields, and those bearing electron-withdrawing furnished desired ketones in moderate yields of 56–62% isolated yields. However, an acid (HCl) is utilized in longer reaction times in this system, and it is thus not green.

**Scheme 34:**
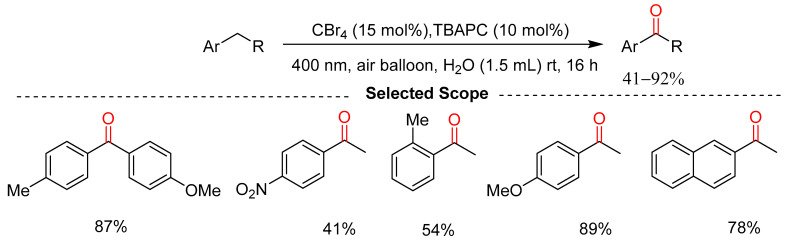
Alkylarene oxidation to ketones reported by Chen and co-workers.

Chen and co-workers documented the photocatalytic oxidation of alkylarenes to carbonyls using carbon tetrabromide in the presence of TBAC in water at room temperature under visible light for 16 h (Scheme 34) [69]. To investigate the substrate scope and limitations, the authors tested their system on diphenylmethane derivates and ethylbenzene derivatives bearing both electron-donating and electron-withdrawing substituents, and the corresponding ketones were obtained in moderate-to-high yields of 37–89%. However, this system was not tested on heterocyclic substrates; therefore, it would be useful to apply this methodology to heterocyclic derivatives as well to determine a broader scope and limitations.

Xu and co-workers reported the selective oxidation of benzylic C–H bonds to carbonyls in the presence of Cs_3_Bi_2_Br_9_/BiOBr as the photocatalyst and molecular oxygen in acetonitrile as the oxidant under irradiation for 4 h using a 300 W Xe lamp, whereby ethylbenzene was converted to acetophenone with a selectivity of 90.4 [70]. Only one example for ketone synthesis was reported as the main focus of the paper was the aldehyde synthesis.

**Scheme 35:**
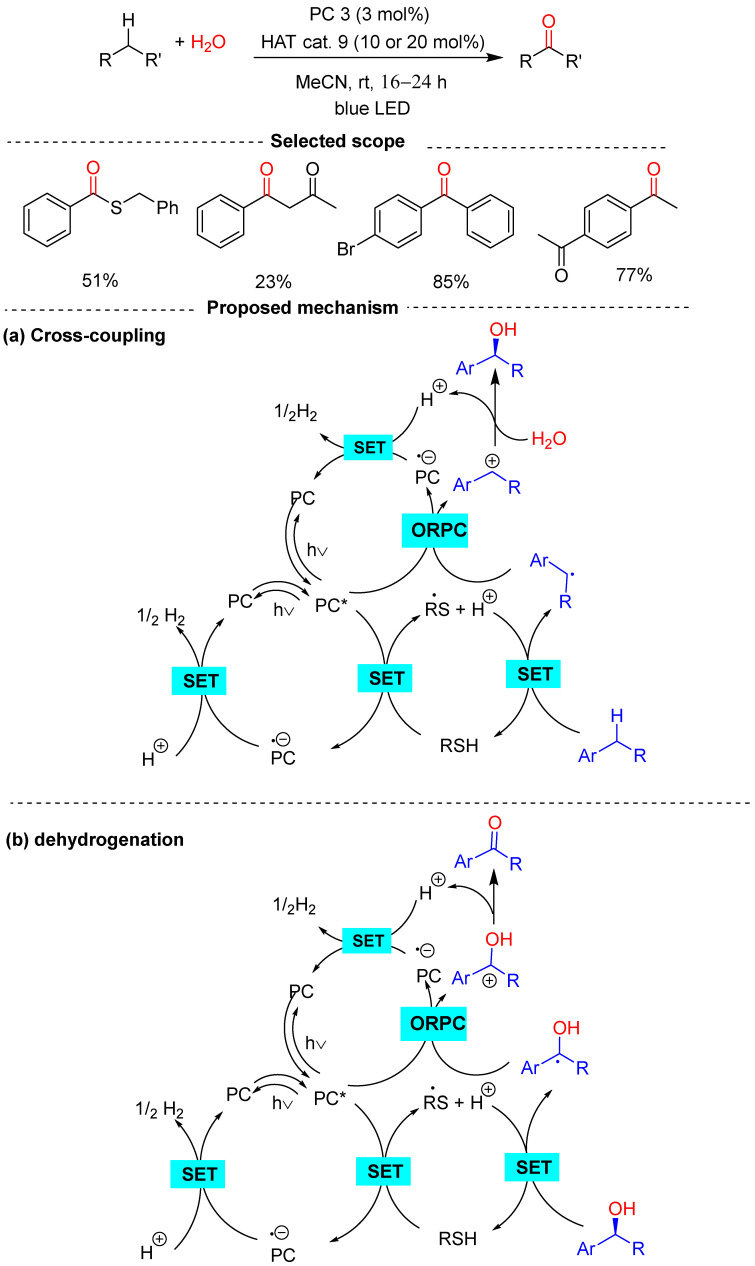
Alkylarene oxidation reported by Guo and co-workers. * indicates activation by light.

Guo and co-workers reported the photocatalytic oxidation of alkylarenes to ketones in the presence of a photocatalyst 4CzIPN (PC 3) and hydrogen atom transfer catalyst ethyl 2-mercaptopropanoate (HAT cat. 9) using water as an oxidant under blue LED light in THF at room temperature for 16 h (Scheme 35) [71]. This system was applicable to a wide range of substrates and the corresponding ketones were obtained in yields of 23–85%. The authors then investigated mechanistics and ran a series of controlled experiments to propose a plausible reaction mechanism that commences with the reductive quenching of the excited catalyst PC by a thiol catalyst via SET to generate the thiyl radical after deprotonation. The alkylbenzene undergoes HAT to the electronic thiyl radical to furnish the nucleophilic benzylic radical, which then undergoes an oxidative radical polar crossover (OPRC) with the excited catalyst PC* forming benzylic cation. H_2_O then captures the cation intermediate to form the cross-coupling product benzyl alcohol after deprotonation. Benzyl alcohol can effectively participate in acceptorless dehydrogenation via another OPRC to give the final ketone.

**Scheme 36:**
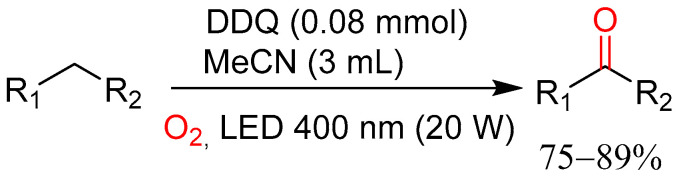
Alkylarene oxidation reported by Kazemi and co-workers.

Recently, Kazemi and co-workers reported the photocatalytic oxidation of benzylic C–H bonds to ketones in the presence of DDQ under visible light in an oxygen-rich environment in acetonitrile at room temperature for 24 h and the corresponding ketones were obtained in good-to-high yields (75–89%) (Scheme 36) [72]. However, only six examples of ketones were synthesized; therefore, it would be useful to test this method on a wide range of substrates to determine its scope and limitations.

### 2.4. Electrochemical Benzylic C–H Oxidation

**Scheme 37:**
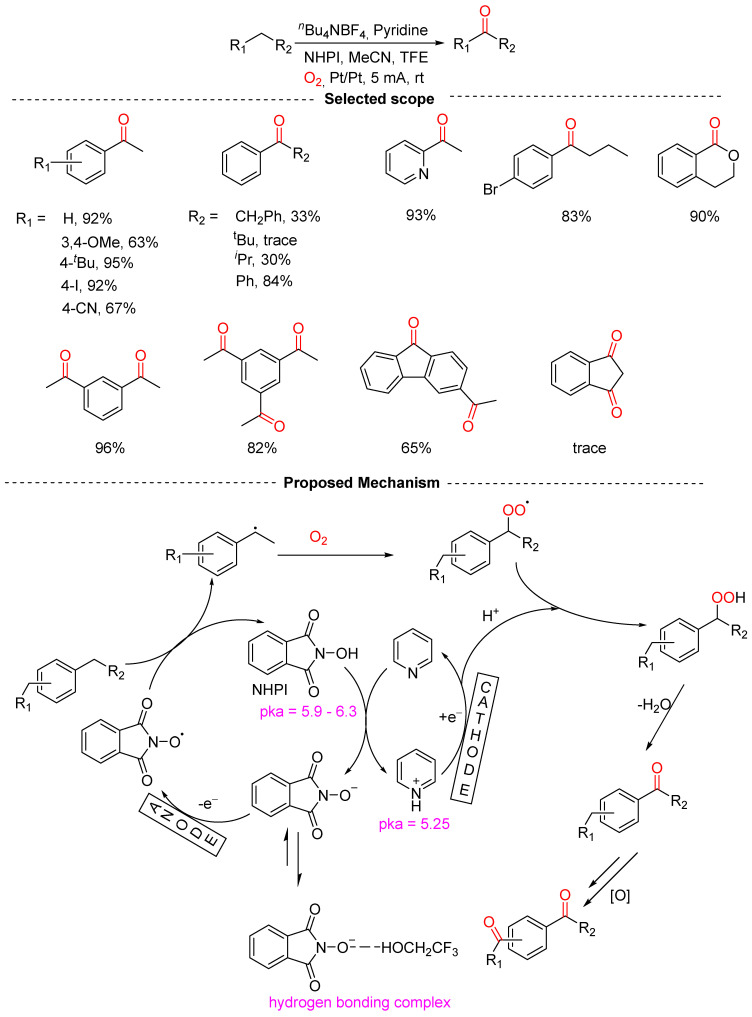
Alkylarene oxidation to aromatic ketones reported by Zha, Wang and co-workers.

Zha, Wang and co-workers reported the selective electrooxidation of aromatic hydrocarbons to aromatic ketones under mild reaction conditions (Scheme 37) [73]. Their system was applicable to a wide range of substrates where those bearing electron-donating and electron-withdrawing substrates afforded the desired products in moderate-to-high yields. Substrates bearing diverse groups on R_2_ afforded the corresponding ketones in moderate-to-high yields (30–84%), except for *tert*-butyl as only trace amounts of the desired product were obtained, due to steric hindrance. The authors also tested this system for multiple oxidation processes, whereby more than one C–H was oxidized to a carbonyl, and the corresponding carbonyls were obtained in moderate-to-good yields (65–96%). However, it was not applicable to 2,3-dihydro-1*H*-indene as the corresponding diketone was obtained in trace amounts. The authors conducted cyclic voltammetry reactions to gain insight into the mechanism and proposed a plausible mechanism. Under electrolysis conditions, an NHPI anion that is generated from pyridine deprotonation is oxidized to the corresponding radical on the surface of the anode. On the other hand, the NHPI anion abstracts a proton from the substrate to generate a benzyl radical which, upon reaction with oxygen followed by reduction, produces a peroxide that undergoes dehydration to furnish the monoketone and further oxidation produces a multiketone.

**Scheme 38:**
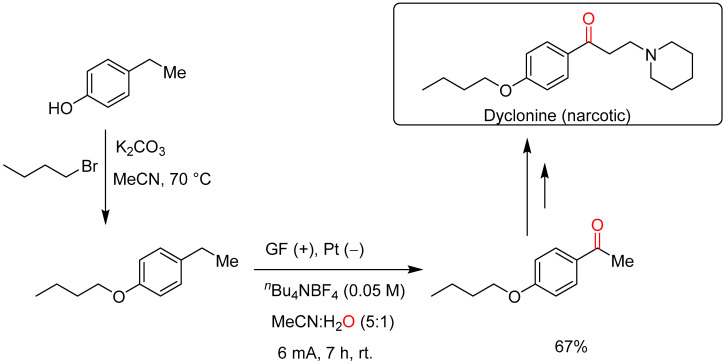
Application of an alkylarene electro-oxidative system to dyclonine intermediate (ketone) reported by Liu and co-workers.

Liu and co-workers reported the electrochemical oxidation of alkylarenes to aromatic ketones using 6 mA constant current at room temperature with a carbon rod as anode and platinum as cathode with tetrabutylammonium tetrafluoroborate as electrolyte. The substrate and scope limitations were investigated where the targeted products were obtained in 11–92% yields. The authors then applied this methodology to prepare the intermediate of dyclonine (Scheme 38) [74]. Some substrates, however, underwent decomposition; hence, lower yields such as 11% were observed.

**Scheme 39:**
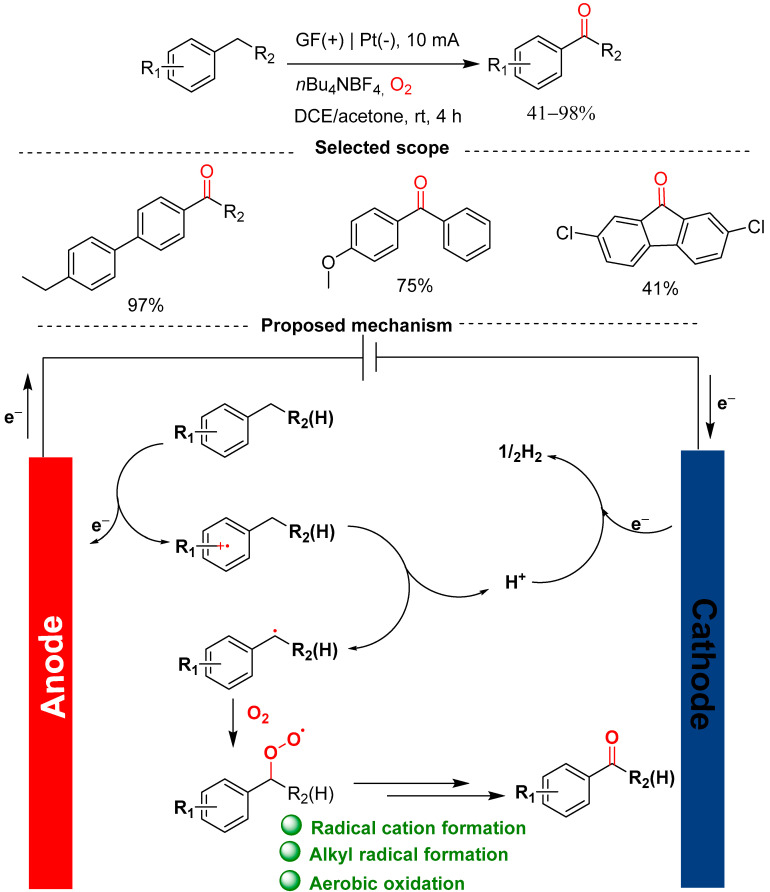
Alkylarene oxidation reported by Bai and co-workers.

Bai and co-workers also reported the electrooxidation of alkylarenes for which they employed the use of DCE/acetone as a solvent, 10 mA, and molecular oxygen as the oxygen source, whereby the corresponding ketones were obtained in moderate-to-excellent yields of 41–98% (Scheme 39) [75]. The authors proposed a reaction mechanism via a single-electron transfer (SET) pathway as a substrate loses an electron at the anode to form a radical cation, which then loses a proton to generate a carbon-centered radical which reacts with molecular oxygen to furnish the corresponding ketone.

**Scheme 40:**
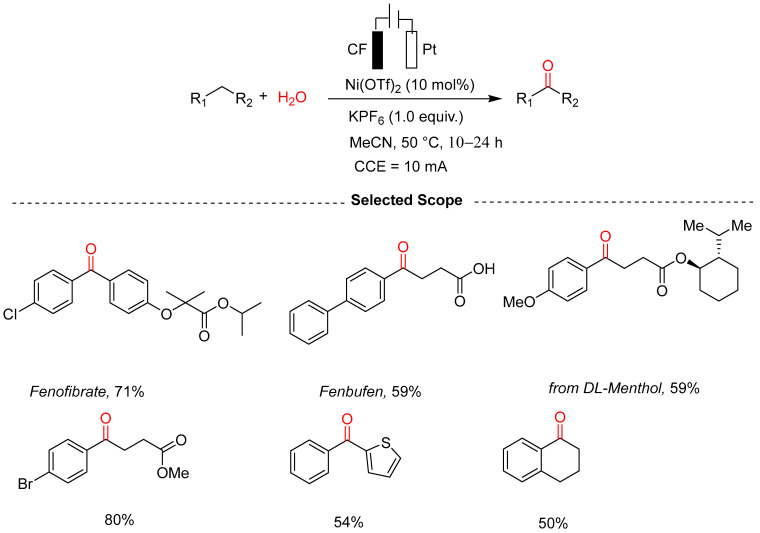
Alkylarene oxidation reported by Zhang and co-workers.

Zhang and co-workers documented the benzylic C–H electrooxidation catalyzed by nickel using carbon felt as the anode, platinum as the cathode in potassium hexafluorophosphate, and acetonitrile at a constant current of 10 mA (Scheme 40) [76]. To determine the scope and limitations, the authors examined the preparation of arenes with functionalized alkyl chains, complex compounds and drug derivatives, functionalized arenes, and diphenylmethane derivatives whereby the target products were obtained in moderate-to-high isolated yields (42–95%). This is a highly desirable system that utilizes earth-abundant metal, nickel, and green solvent, water, as the oxygen source. It was applicable to a broader substrate scope such that even alkylarenes derived from drugs such as DL-Menthol were successfully oxidized to ketones in moderate yields to excellent yields of 48–95%.

**Scheme 41:**
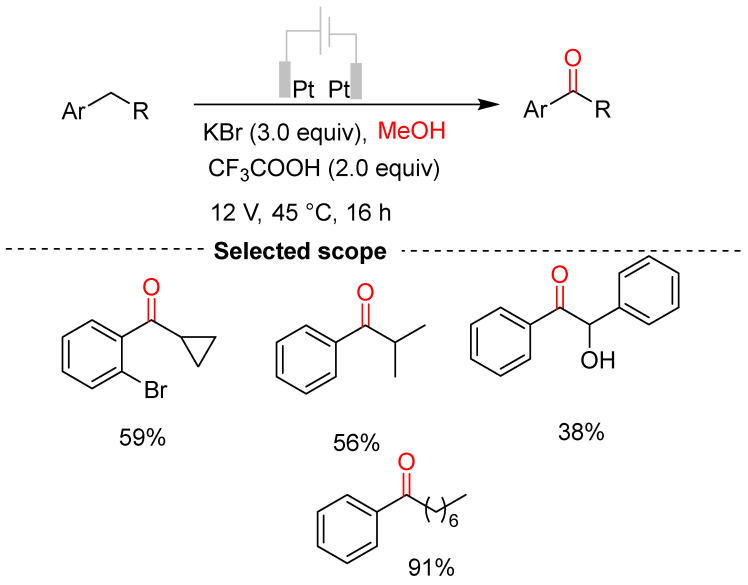
Alkylarene oxidation reported by Zhao, Zhou, Zhang and co-workers.

Zhao, Zhou, Zhang and co-workers also reported electrochemical benzylic C–H oxidation of alkylarenes to aromatic ketones, except platinum was used as both the anode and the cathode, methanol as an oxidant, and potassium bromide as both the electrolyte and a bromide radical precursor in trifluoroacetic acid at 12 V constant voltage for 16 h (Scheme 41) [77]. The substrate and scope limitations were analyzed, and the corresponding ketones were obtained in 21–91% yields. In this system, multi-oxidation was also observed as the authors were able to synthesize diketones from alkylarenes.

**Scheme 42:**
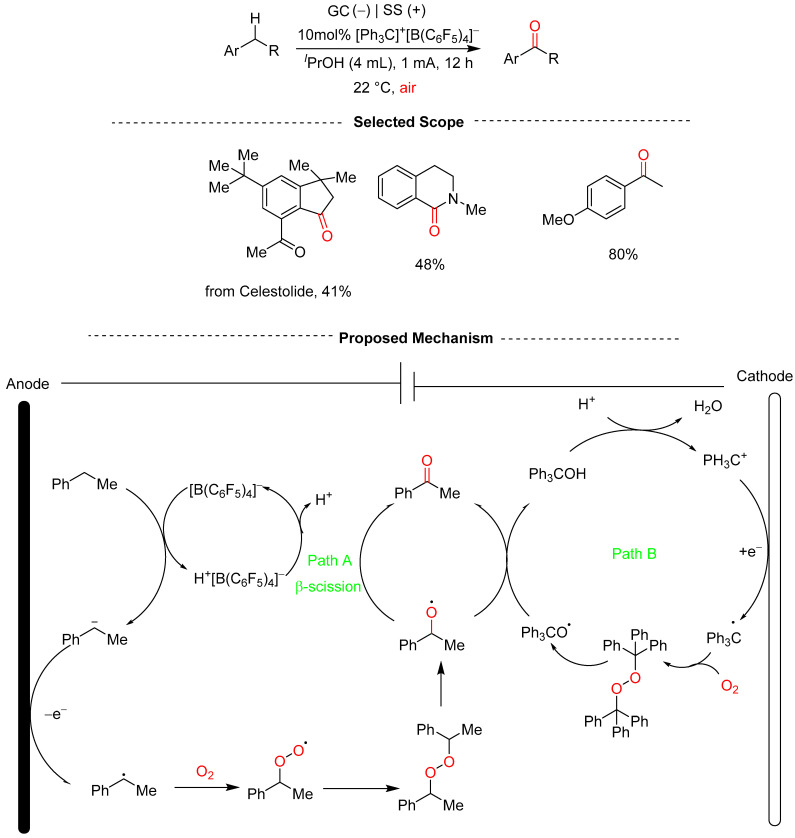
Alkylarene oxidation reported by Jia and co-workers.

Jia and co-workers reported the triaryl carbenium ion pair-mediated electrocatalytic benzylic C–H oxygenation of alkylarenes to aromatic ketones in air. Trityl tetrakis-(pentafluorophenyl)-borate ([Ph_3_C]^+^[B(C_6_F_5_)_4_]^−^) was used as a catalyst and electrolyte, GC as the anode, and SS as the cathode in isopropanol at 1 mA constant current conducted under open air (Scheme 42) [78]. This system was applicable to a broad substrate scope and the corresponding ketones were obtained in moderate-to-high yields (41–80%). The authors proposed a reaction mechanism after a series of control experiments. The substrate is deprotonated by the [B(C_6_F_5_)_4_]^−^ anion to generate a benzylic anion following single-electron oxidation by the anode to form the benzylic radical which, upon reaction with oxygen, afforded the peroxide radical which underwent coupling with the benzylic radical to form the peroxide intermediate that undergoes homolysis to form a reactive oxygen centered radical that undergoes *β*-scission to form the product (Path A). Meanwhile, the triphenylcarbenium cation was reduced by the cathode to form the trityl radical, which coupled with oxygen to generate the corresponding peroxide intermediate. Another reactive oxygen-centered radical was generated in situ via homolysis and abstracted a proton to generate a ketone and triphenylmethanol which undergoes protonolysis to recycle a triaryl carbenium (Path B). This methodology is the most sustainable and cost-effective because it works in the absence of an external supporting electrolyte and uses an inexpensive oxidant, molecular oxygen.

**Scheme 43:**
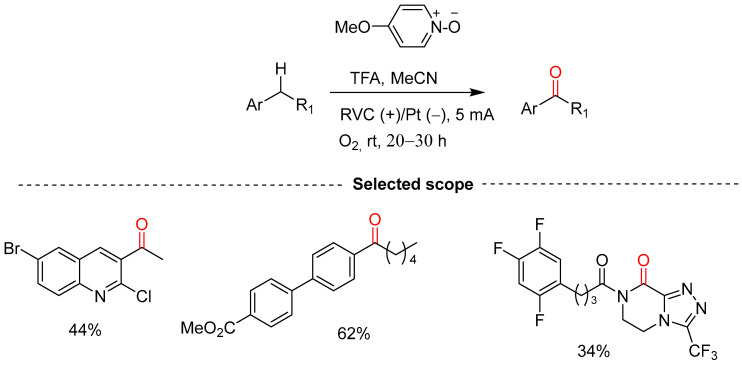
Alkylarene oxidation reported by Shen, Ye and co-workers.

Recently, Shen, Ye and co-workers reported the pyridine *N*-oxide-mediated electrochemical benzylic C–H oxygenation of alkylarenes to ketones which was applicable to a broader substrate scope with corresponding ketones obtained in moderate-to-high yields (48–97%) (Scheme 43) [79]. Although this system works at room temperature, it is disadvantaged by prolonged reaction times of 20–30 h.

## 3. Applications of Benzylic C–H Oxidation in Heterocyclic Compounds Preparation

Heterocycles are compounds containing at least one heteroatom such as nitrogen, oxygen, sulfur, etc., found in many natural products, drugs, and biologically active compounds such as anticancer, antidepressant, and antibiotics [80,81,82]. Nitrogen-containing heterocycles such as quinoxalines, imidazoles, and oxazoles play an important role in biological and pharmaceutical chemistry [83]. The synthesis of heterocyclic compounds has gained a considerable amount of interest because of their extensive application in materials science and bioactive molecules [84].

### 3.1. Six-Membered Heterocycles

**Figure 3:**
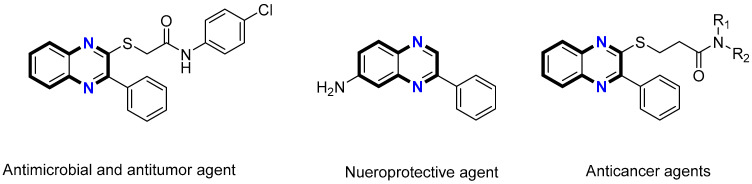
Biologically important quinoxalines.

**Scheme 44:**
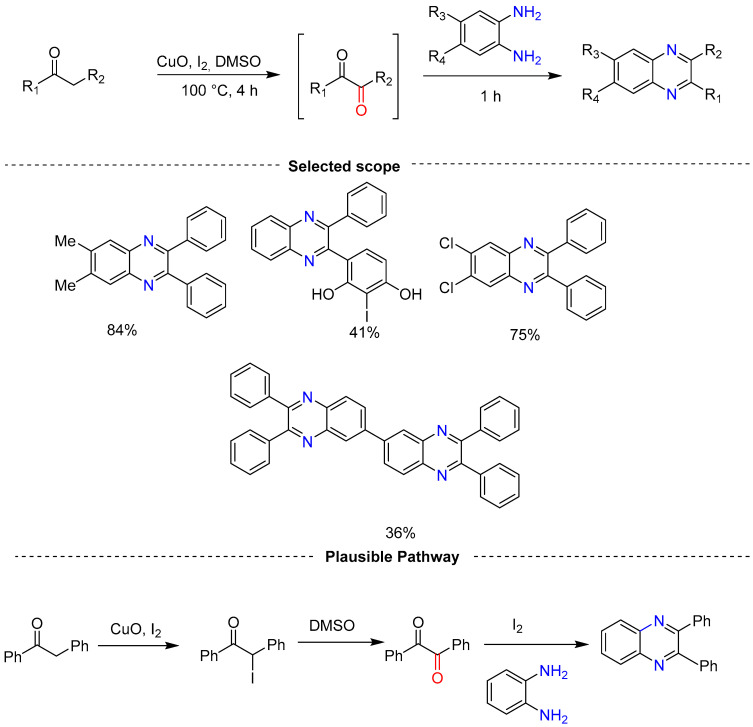
Quinoxaline synthesis reported by Yin and co-workers.

Quinoxalines are six-membered heterocycles with two nitrogen atoms found in various natural products with useful applications ranging from biologically important, i.e., anticancer [85,86], to materials, i.e., fluorescent sensors [87]. Some examples of biologically important quinoxalenes are shown in Figure 3. Yin and co-workers reported the preparation of quinoxalines from α-methylene ketones and phenyldiamines in the presence of CuO, molecular oxygen in DMSO, and the quinoxaline derivatives were obtained in moderate-to-good yields (41–84%). The authors proposed a plausible reaction pathway commencing from the iodination of the substrate using molecular iodine and cupric oxide followed by oxidation to form 1,2-diketone in the presence of DMSO. The in situ generated diketone then cyclizes with the diamine to furnish the desired quinoxaline (Scheme 44) [88].

**Scheme 45:**
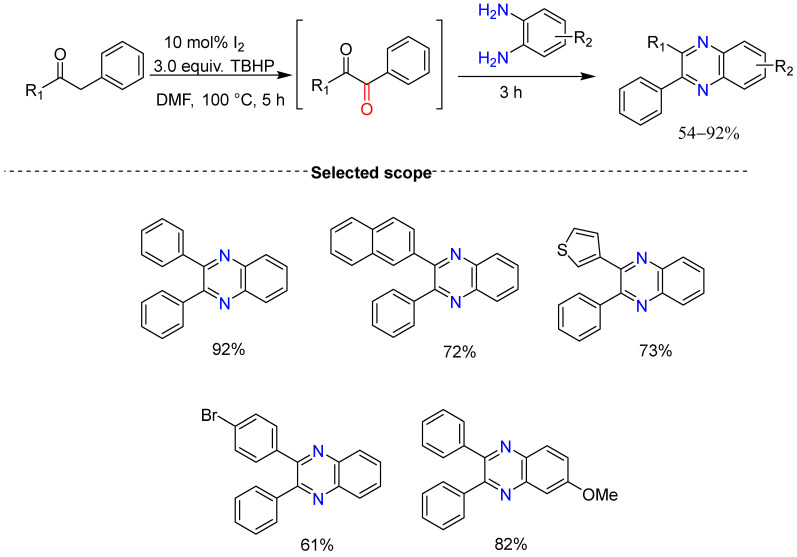
Synthesis of quinoxalines reported by Li, Bai and co-workers.

Li, Bai and co-workers reported the one-pot synthesis of quinoxalines from α-methylene ketones and *o*-phenylenediamines catalyzed by molecular iodine. The α-methylene ketone was oxidized to 1,2-diketones by TBHP in the presence of molecular iodine in DMF at 100 °C for 5 h, and the in situ generated ketone was then coupled with phenyldiamine to furnish the desired quinoxaline (Scheme 45) [89]. This methodology was applicable to a wide range of substrates as about 17 examples were synthesized in moderate-to-good yields (54–92%). However, the use of stochiometric amounts of TBHP and a non-green solvent DMF detracts from this method [90].

**Scheme 46:**
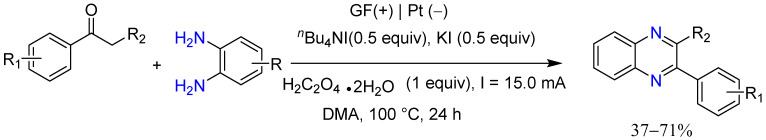
Synthesis of quinoxalines via benzylic C–H oxidation reported by Liu and co-workers.

Recently, Liu and co-workers reported the electrosynthesis of quinoxalines from ketones and *o*-phenyldiamines catalyzed by iodine where GF was used as the anode and Pt as the cathode (Scheme 46) [91]. The substrate and scope were investigated, and the corresponding quinoxalines were obtained in moderate yields of 35–71%.

### 3.2. Five-Membered Ring Heterocycles

**Figure 4:**
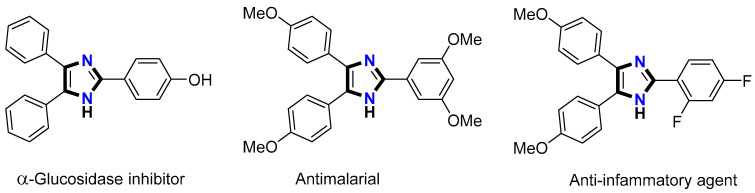
Examples of biologically important 2,4,5-trisubstituted imidazoles.

**Scheme 47:**
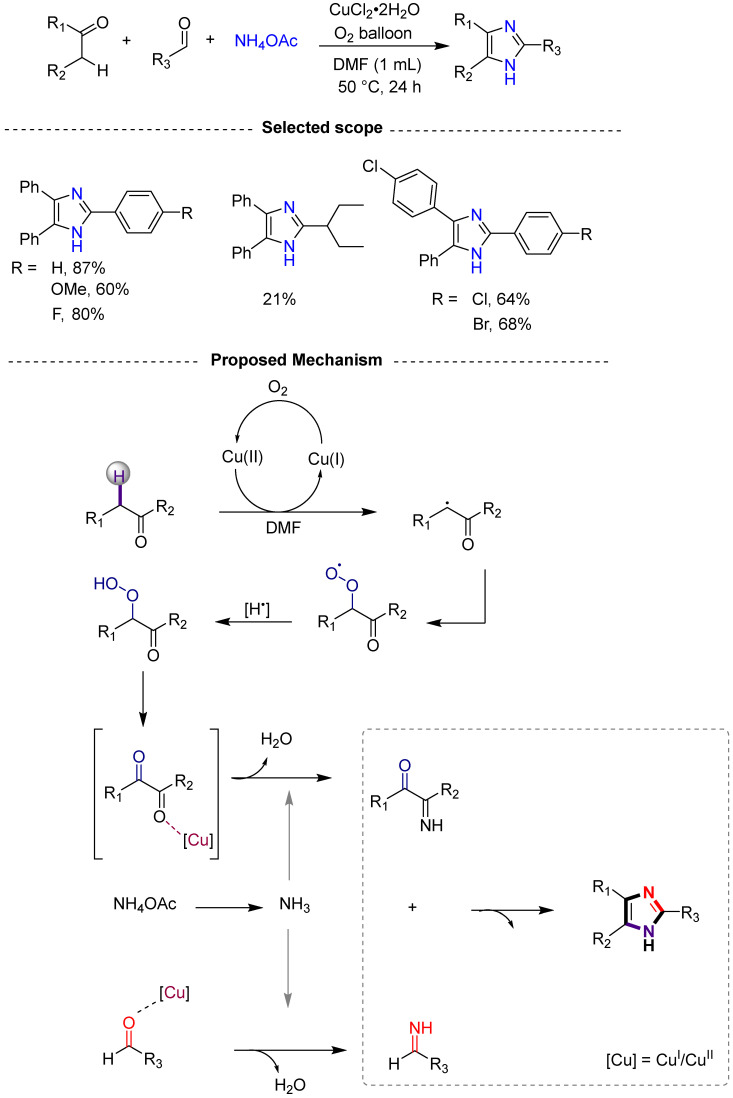
Imidazole synthesis reported by Jayram and Jeena.

Imidazoles are fundamental five-membered heterocycles found in several naturally occurring compounds such as biotin and histamine. 2,4,5-Trisubstituted imidazoles have been shown to exhibit various biological activities such as antidiabetic [92], anti-inflammatory [93], antibacterial [94], and antimalarial [95] (Figure 4). These fascinating compounds are typically prepared by the cyclocondensation reaction between an α-diketone, an aldehyde and ammonium acetate. Our research group documented the multicomponent synthesis of 2,4,5-trisubstituted imidazoles from α-methylene ketones, aldehydes, and ammonium acetate in the presence of CuCl_2_·H_2_O and molecular oxygen in DMF at 50 °C for 24 h (Scheme 47) [96]. The analysis of substrate and scope limitations was conducted and the corresponding trisubstituted imidazoles were obtained in yields of 21–87%. We then proposed a reaction mechanism which commences from the oxidation of α-methylene ketone by Cu(II) and deprotonation to form benzyl radical which is then trapped by oxygen to generate a peroxy radical. The hydrogen radical is then captured by a peroxy radical followed by dehydration to furnish the 1,2-diketone. Subsequently, an aldehyde and 1,2-diketone are activated by Cu species to form imine intermediates which, upon reaction with ammonia, cyclocondense to furnish the trisubstituted imidazoles.

**Scheme 48:**
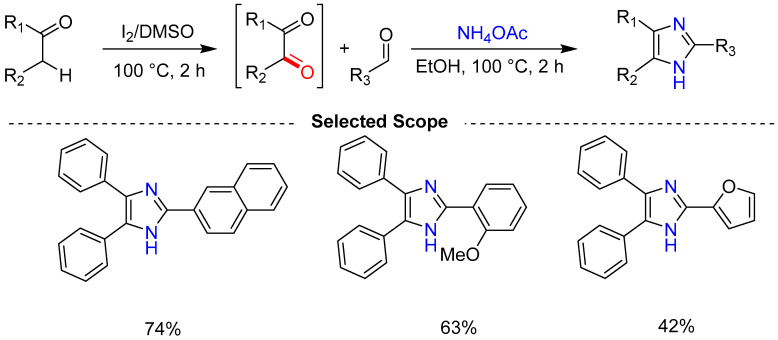
Synthesis of 2,4,5-trisubstituted imidazoles reported by Jayram and Jeena.

Due to our continuous interest in heterocyclic synthesis, in 2019 we reported the synthesis of 2,4,5-trisubtituted imidazoles using the I_2_/DMSO system from α-methylene ketones (Scheme 48) and the corresponding 2,4,5-trisubstituted imidazoles were obtained in moderate-to-good yields (35–86%) [97]. This method was applicable to a wide substrate scope including aliphatic, heterocyclic, and bulkier substituents. In 2022, we followed up with a Domino convergent synthesis of 2,4,5-trisubstituted imidazoles via the dual oxidation of α-methylene ketones and alcohols using the I_2_/DMSO system [98]. This methodology was applicable to a wide range of substrate scope and the targetedtrisubstituted imidazoles were obtained in moderate-to-high yields (50–84%).

**Figure 5:**
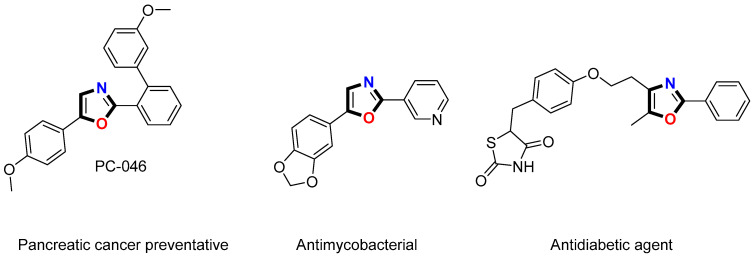
Examples of biologically important oxazoles.

**Scheme 49:**
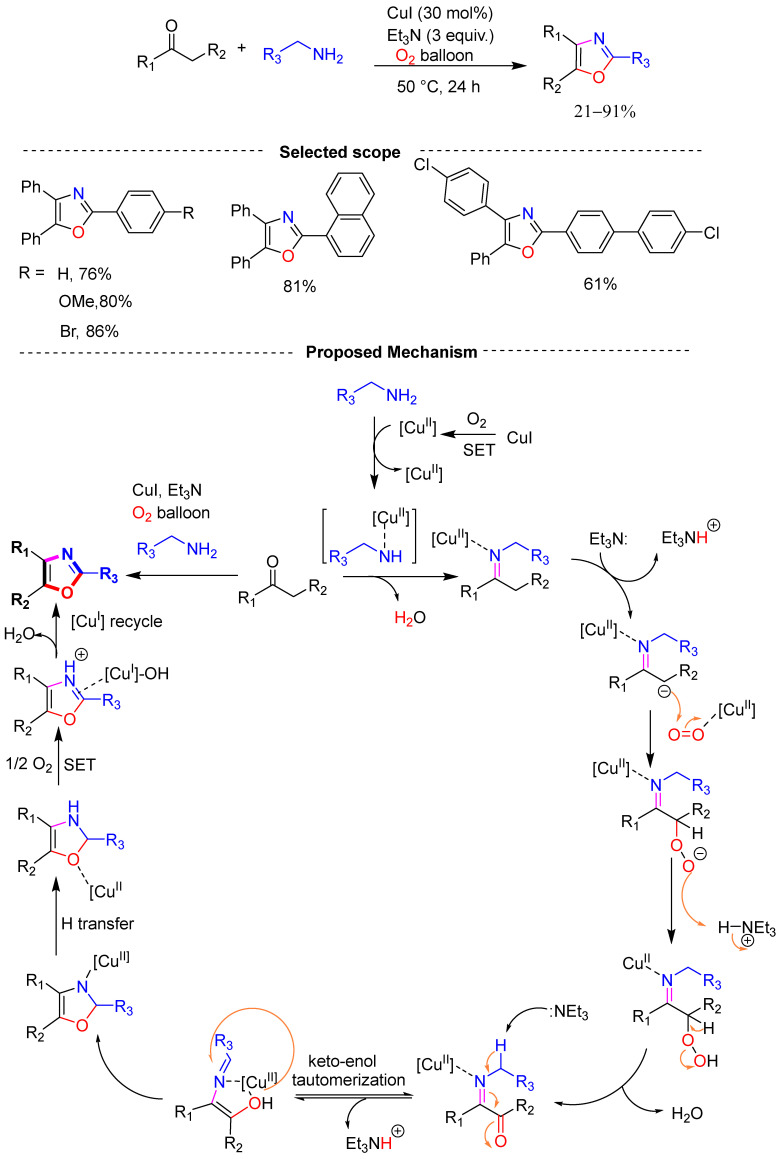
Synthesis of oxazole reported by Mazibuko and Jeena.

Oxazoles are among the most vital heterocyclic organic scaffolds found in various biologically active compounds [99]. 2,4,5-Trisubstituted oxazoles have been shown to have several pharmacological properties such as antiobesity [100], antineuropathic [101], and antitubercular [102]. The selected examples in Figure 5 include pancreatic cancer preventative [103], antimycobacterial [104,105], and antidiabetic [106]. Our research group reported the benzylic C–H aerobic oxidative annulation synthesis of oxazoles from the benzylic oxidation of α-methylene ketone and amines in the presence of copper iodide (Scheme 49) [107]. This methodology was applicable to a broad range of substrates and the corresponding oxazoles were obtained in 21–91% yields. After a series of control experiments, a plausible reaction mechanism was proposed which commences by a generation of [Cu^II^] from Cu^I^ and O_2_ through a single electron transfer (SET) which then facilitates the amino proton removal from the amine, thus activating the [Cu^II^]- amino complex that reacts with a methyl ketone via a nucleophilic attack targeting the carbonyl carbon to produce an intermediate. The β-carbon of the intermediate is then deprotonated by Et_3_N to furnish the carbanion intermediate which is then oxidized by O_2_/[Cu^II^] to generate the peroxide intermediate that abstracts hydrogen from a protonated Et_3_N to form a hydroperoxide intermediate which undergoes dehydration to form a key intermediate. The keto-enolization then forms the enol isomer and intramolecular cyclization, intramolecular hydrogen transfer, and a reaction with half an oxygen molecule results in an imine-type intermediate. [Cu^II^] becomes [Cu^I^] through SET and subsequent dehydration results in the recycling of [Cu^I^] and furnishes the desired oxazole.

## 4. Concluding Remarks

The benzylic C–H oxidation of alkylarenes to aromatic ketones is a transformation that plays a vital role in organic synthesis and pharmaceutical chemistry. As demonstrated from the literature analysis, significant progress has been made in the reviewed field for the past five years. Various research groups have explored this ever-emerging transformation for the selective benzylic C–H oxidation of alkylarenes to aromatic ketones, where transition-metal catalysis was predominant. Inexpensive and environmentally benign molecular oxygen was increasingly used as an oxidant in these reactions. Furthermore, photocatalytic and electrochemical strategies are progressively being applied in this transformation. The major challenges associated with benzylic C–H oxidation strategies include the occurrence of side products, longer reaction times, and the use of expensive metals such as ruthenium and commercially unavailable catalysts that must be prepared at higher temperatures. In other systems, the scope and limitations were not properly determined as a narrow range of substrates were investigated. A solution to the above-mentioned challenges would be the development of highly selective, inexpensive, and environmentally benign benzylic C–H oxidation systems that may pioneer novel approaches to complex molecules in synthetic organic chemistry and pharmaceuticals. The most significant recent advance is the metal- and oxidant-free carbonylation of benzylic and allylic C–H bonds via dual oxidative and radical–polar crossover which utilizes water as an oxidant, applicable to a broad substrate scope under mild conditions [70]. The use of water as an oxidant is particularly attractive from a green chemistry perspective owing to its environmental friendliness. Additionally, we have covered the application of benzylic C–H oxidation as a key step in the preparation of quinoxalines, imidazoles, and oxazoles from methylene ketones. The above-mentioned approaches can potentially be utilized in synthesizing novel biologically active aromatic ketones and heterocyclic compounds.

## Figures and Tables

**Table 1:**
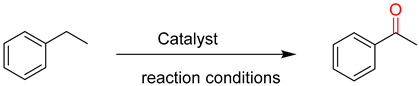
Oxidation of ethylbenzene to acetophenone using different catalysts.

Entry	Catalyst	Reaction Conditions	%Conv.	%Select.	Ref.
1	Co/Ph-HMS	O_2_ (1.0 MPa), 6 h, 393 K	55.1	86.1	Shi and co-workers [29]
2	*N*-hydroxy-1,6-methano [10]-annulene-3,4-dicarboximide/Co(OAc)_2_	O_2_ (0.1 MPa) in AcOH, 6 h, 100 °C	75	63	Zuo and co-workers [30]
3	Co-Cu/SAPS-15	TBHP, 6 h, 100 °C	96.54	99.77	Chaudhary and Sharma [31]
4	Pd/CeO_2_ (10 wt%)	TBHP (1 mmol), 4 h, 80 °C	99.8	79	Kalita and Saikia [32]
5	Mn-ZSM-5-50	TBHP (3 mmol), 6 h, 80 °C	43.6	68.8	Liu and co-workers [33]

## Abbreviations

atm	atmospheres
Ar	Argon
AQ	Anthraquinone
BQC	2,2′-biquinoline-4,4′-dicarboxylic acid dipotassium salt
BiOBr	Bismuth bromide oxide
CBr_4_	Tetrabromomethane
CeO_2_	Ceric oxide
CF	Carbon felt
CF_3_SO_2_Na	trifluoromethanesulfinate
Co	Cobalt
Co(OAc)_2_	Cobalt (II) acetate
Conv.	Conversion
Cu	Copper
CuCl_2_	Copper (II) Chloride
Cu_2_O	Copper (I) oxide
CuO	Copper (II) oxide
CuI	Copper iodide
Cu_2_(O_2_CR)_4_	Copper (II) carboxylate
CuOTf	Copper triflate
Cr	Chromium
CPET	Concerted proton–electron transfer
DDQ	2,3-dichloro-5,6-dicyano-1,4-benzoquinone
DMF	Dimethyl formamide
DMSO	Dimethyl sulfoxide
DTBP	Di
EtOH	Ethanol
EPR	Electro paramagnetic resonance
Fe(NO_3_)_3_·9H_2_O	Iron (III) nitrate nonahydrate
GC	Glassy Carbon
GF	Graphite Felt
GVL	-valerolactone
h	hours
HAT	Hydrogen atom transfer
HCl	Hydrochloric acid
H_2_C_2_O_4_·H_2_O	Oxalic acid dihydrate
H_2_O	Water
H_2_O_2_	Hydrogen peroxide
HMS	Hexagonal mesoporous silica
K	Kelvin
K_2_CO_3_	Potassium carbonate
KPF_6_	Potassium hexafluorophosphate
KI	Potassium iodide
LaMnO_3_	Lanthanium manganite
LED	Light-emitting diode
MeCN	Acetonitrile
Mg_6_MnO_8_	Murchodite-type oxide
MgFe-LDH	Magnesium-iron layered double hydroxide
MFM-170	[Cu_2_(L)](H_4_L = 4′, 4′′′-(pyridine-3,5-diyl)bis([1,1′-biphenyl]-3,5-dicarboxylic acid
mL	milliliters
Mn	Manganese
Mn-ZSM-5-50	Manganese-incorporated ZSM-5 zeolite
MOFs	Metal–organic frameworks
MPa	Megapascals
Na	Sodium
Na_2_CO_3_	Sodium carbonate
NBu_4_Cl	Tetrabutylammonium chloride
*^n^*Bu_4_NI	Tetrabutyl ammonium iodide
NFSI	N-fluorobenzenesulfonimide
NHI	N-Hydroxyimide
NHPI	N-hydroxyphthalimide
(NH_4_)_3_[CrMo_6_O_18_(OH)_6_]	Ammonium Hexamolybdochromate(III)
Ni(OTf)_2_	Nickel (II) trifluoromethanesulfonate
Ni_2_Mn-LDH	Nickel-manganese layered double hydroxide
nm	Nanometers
O_2_	Molecular oxygen
PC-4	6-Bromobenzo[*d*]thiazole
Pd	Palladium
PhCN	Benzonitrile
[Ph_3_C]^+^[B(C_6_F_5_)_4_]^−^	Triphenylmethyliumtetrakis(perfluorophenyl)borate
POMOFs	Polyoxometalate-based metal–organic frameworks
*^i^*PrOH	Isopropyl alcohol
Pt	Platinum
PT-ET	Proton-transfer/electron-transfer
rpm	Rotations per minute
rt	Room temperature
RuO_2_	Ruthenium dioxide
Select.	Selectivity
SPXRD	Synchroton powder X-ray diffraction
SS	Stainless steel
T(2,3,6-triCl)PPCo	5,10,15,20-Tetrakis(2,3,6-trichlorophenyl)porphyrin Cobalt (II)
TBAC	Tetrabutylammonium chloride
TBHP	*tert*-Butyl Hydroperoxide
TEMPO	2,2,6,6-tetramethyl,1-piperidonyloxy
TiO_2_	Titanium dioxide
UV	Ultraviolet
UVA	Ultraviolet A
UVB	Ultraviolet B
V_2_O_5_	Vanadium (V) pentoxide
